# Four ways of implementing robustness quantification in strain characterisation

**DOI:** 10.1186/s13068-023-02445-6

**Published:** 2023-12-19

**Authors:** Luca Torello Pianale, Fabio Caputo, Lisbeth Olsson

**Affiliations:** Industrial Biotechnology Division, Department of Life Sciences, Chalmers University of Technology, Gothenburg, Sweden

**Keywords:** *Saccharomyces cerevisiae*, Yeast, Biosensors, Bioprocess, Intracellular environment, Physiology

## Abstract

**Background:**

In industrial bioprocesses, microorganisms are generally selected based on performance, whereas robustness, i.e., the ability of a system to maintain a stable performance, has been overlooked due to the challenges in its quantification and implementation into routine experimental procedures. This work presents four ways of implementing robustness quantification during strain characterisation. One *Saccharomyces cerevisiae* laboratory strain (CEN.PK113-7D) and two industrial strains (Ethanol Red and PE2) grown in seven different lignocellulosic hydrolysates were assessed for growth-related functions (specific growth rate, product yields, etc.) and eight intracellular parameters (using fluorescent biosensors).

**Results:**

Using flasks and high-throughput experimental setups, robustness was quantified in relation to: (i) stability of growth functions in response to the seven hydrolysates; (ii) stability of growth functions across different strains to establish the impact of perturbations on yeast metabolism; (iii) stability of intracellular parameters over time; (iv) stability of intracellular parameters within a cell population to indirectly quantify population heterogeneity. Ethanol Red was the best-performing strain under all tested conditions, achieving the highest growth function robustness. PE2 displayed the highest population heterogeneity. Moreover, the intracellular environment varied in response to non-woody or woody lignocellulosic hydrolysates, manifesting increased oxidative stress and unfolded protein response, respectively.

**Conclusions:**

Robustness quantification is a powerful tool for strain characterisation as it offers novel information on physiological and biochemical parameters. Owing to the flexibility of the robustness quantification method, its implementation was successfully validated at single-cell as well as high-throughput levels, showcasing its versatility and potential for several applications.

**Graphical Abstract:**

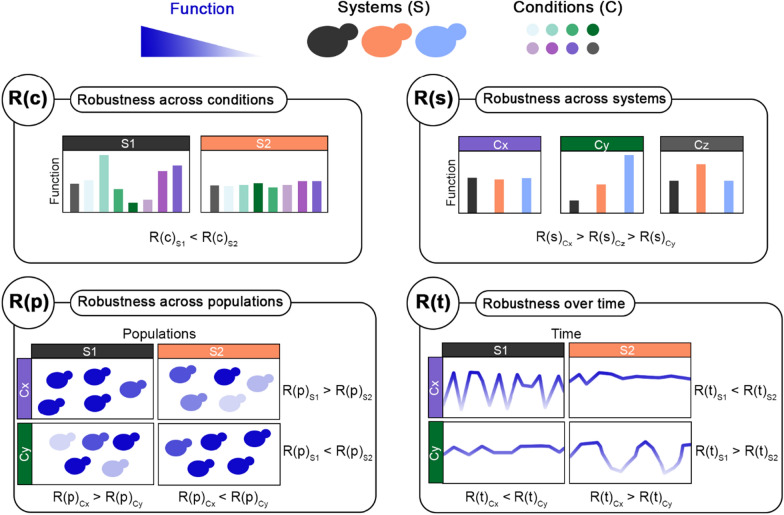

**Supplementary Information:**

The online version contains supplementary material available at 10.1186/s13068-023-02445-6.

## Background

Industrial bioprocesses employ microorganisms for sustainable large-scale production of a broad range of chemicals used in pharmaceuticals, agriculture, biofuels, biochemicals and bioplastics. Although industries generally select their production hosts based on output, microorganisms with reliable and stable production performance (e.g., product titers, rates and yields) are often preferred. The feature for which a microorganism can perform in a stable way across different perturbations is referred to as “robustness” and can be crucial when selecting and improving microorganisms for bioproduction [1]. However, assessing robustness during strain screening, selection, and development has often been neglected due to difficulties in its quantification. Recently, a Fano factor-based, dimensionless, free-from-arbitrary control conditions and frequency-independent robustness quantification method has been developed [2]. This method (i.e., Trivellin’s formula) allowed the identification of robust functions (e.g., specific growth rate or product yields) among the tested strains, as well as performance-robustness trade-offs in a perturbation space composed of single stress conditions coming from the lignocellulosic hydrolysate perturbation space (featuring inhibitory compounds, osmotic stress, and product inhibition, etc.) [2, 3]. Trivellin’s robustness equation highlighted how robustness computed with this formula is a relative (not absolute) feature of functions (such as specific growth rate, product yields, etc.) with respect to the systems (strains) considered [1, 2]. Moreover, as the Fano factor itself is generally used to assess the dispersion of data for the functions considered [4], Trivellin’s robustness equation can be potentially applied to answer a wide range of questions regarding function stability in a broader sense.

Lignocellulosic hydrolysates are complex substrates rich in various sugars and are derived from pre-treated plant biomass, such as wood waste or side streams. They are used for second-generation (2G) biofuel production, an environmentally friendly alternative to fossil fuels [5]. These substrates are fermented by microorganisms, including the yeast *Saccharomyces cerevisiae*, which is a widely employed and efficient host [6]. However, 2G biofuel production still faces challenges related to cost-effectiveness, scalability, and fermentability of hydrolysates [7, 8]. Due to the disparate origin of plant biomass, the composition of lignocellulosic hydrolysates varies in terms of the sugars and inhibitory compounds released during pre-treatment [9, 10]. Often, the effect of inhibitors on microbial growth and metabolism is assessed by adding one or a few of them at a time, losing information on the substrate complexity and compound synergy, which play a crucial role in affecting microbes’ performance.

Investigation of the intracellular environment during bioprocesses remains limited by the lack of tools for real-time monitoring. The *Sc*EnSor Kit enables the monitoring of eight intracellular parameters using fluorescent biosensors [11, 12]. While this kit has already been used for studies in a defined synthetic hydrolysate [11], it has never been applied with real lignocellulosic hydrolysates or for robustness analysis. The *Sc*EnSor Kit is suitable for investigating individual cells and populations, making it an optimal tool to investigate population heterogeneity. Subpopulations with different phenotypes are generally present within the same bulk isogenic population and its occurrence poses a problem in industrial settings as it may cause decreased production yields [1, 13]. Thus, having a tool to explore, quantify and evaluate population heterogeneity at an early stage of strain development and selection would be highly desirable.

This study aimed to showcase four simple means of implementing robustness analysis in the physiological characterisation of three *S. cerevisiae* strains (the laboratory CEN.PK113-7D strain, and the industrial Ethanol Red and PE2 strains) in seven lignocellulosic hydrolysates. To this end, growth-related functions, such as specific growth rate and product yields, and eight intracellular parameters measured via fluorescent biosensors from the *Sc*EnSor kit [12] were determined. Three experimental setups, including aerobic and anaerobic flask screenings and high-throughput screening, were employed. As the robustness quantification method previously developed was based on the Fano factor [2], the concept of robustness was here broadened to assess the stability of functions in four different ways. Trivellin’s robustness equation was, therefore, used to assess: (i) stability of growth functions for each strain across different hydrolysates; (ii) similarity of growth functions across strains in each hydrolysate to establish the impact of hydrolysates on yeast metabolism; (iii) dispersion of intracellular parameters over time with respect to their mean, to reveal their stability for each strain and hydrolysate; and (iv) homogeneity of intracellular parameters in a cell population, to indirectly quantify population heterogeneity. Robustness quantification is a powerful tool which provides new perspectives on strain characterisation for academic and industrial purposes. Due to the flexibility of the robustness quantification formula, very different research questions can be accommodated, showing the potential to be applied in many branches of biotechnology.

## Materials and methods

### Strains and media

The *S. cerevisiae* strains used in this study were CEN.PK113-7D (MATa URA3 HIS3 LEU2 TRP1 MAL2-8c SUC2) [14], as well as bioethanol-producing Ethanol Red (Société Industrielle Lesaffre, Division Leaf) and PE2 [15]. Biosensors were integrated into the genome of all strains using the *Sc*EnSor kit [12] (Addgene repository ID #1000000215). The biosensors provided a means of monitoring intracellular pH [16], intracellular ATP [17], glycolytic flux [18], oxidative stress (OxSR) [19], unfolded protein response (UPR) [20], ribosome abundance [11], pyruvate metabolism, and ethanol consumption [12].

Synthetic-defined minimal Verduyn (“Delft”) medium adjusted to pH 5 was used as control medium. It contained 20 g/L glucose, 5 g/L (NH_4_)_2_SO_4_, 3 g/L KH_2_PO_4_, 1 g/L MgSO_4_ · 7H_2_O, 20.4 g/L K-phthalate, 1 mL/L trace metal solution, and 1 mL/L vitamin solution [11]. Vitamin and trace metal solution compositions can be found in Additional file 1: Table S1 (Additional File 1). The composition of lignocellulosic hydrolysates is listed in Table 1; the undiluted hydrolysate was set to 100%. Christian Roslander and Mats Galbe at Lund University, Sweden, carried out pre-treatment of raw plant biomass and enzymatic hydrolysis to produce the hydrolysates (Table 2), which were then diluted to obtain the desired percentage (60% vol/vol for BioLector I screening and 50% vol/vol for flasks). The hydrolysates were supplemented with 5 g/L (NH_4_)_2_SO_4_, 3 g/L KH_2_PO_4_, 1 g/L MgSO_4_ · 7H_2_O, 20.4 g/L K-phthalate, 1 mL/L trace metal solution, and 1 mL/L vitamin solution. The final pH was adjusted to 5. Prior to use, all hydrolysates were filter-sterilised with Whatman^®^ paper to remove most solids and 0.2 μm aPES to ensure sterility.

**Table 1:** Composition of the lignocellulosic hydrolysates used in this study

Compound	Wheat straw^a^	Sugarcane bagasse^a^	Corn stover^a^	Oat hulls^a^	Birch^b^	High-gravity spruce^b^	Softwood logging residues^b^
Glucose	82.3	95.6	62.5	37	124.4	84.9	26.7
Mannose	–	–	–	–	3.2	31.6	21.6
Galactose	1.3	0.7	1.3	2.6	1.7	6.2	9.4
Arabinose	3.1	2	1.9	5.3	1.1	4	4.3
Xylose	38.1	47.1	26.3	87.7	63.8	15	16.5
Formic acid	0.2	–	–	3.1	1.5	1.4	0.1
Acetic acid	5	4.7	2.5	5.1	13.4	7.8	2.7
Levulinic acid	2.1	–	–	–	–	3.6	0.7
Furfural	3.2	1.1	2.7	3.4	0.6	0.4	0.5
Hydroxymethylfurfural	–	–	0.4	–	–	0.9	0.7

Concentrations are expressed in g/L and refer to the pure hydrolysate (100%)

^a^ Non-woody hydrolysates. ^b^ Woody hydrolysates

**Table 2:** Pre-treatment conditions employed to obtain lignocellulosic hydrolysates

Raw material	Pre-treatment
Oat hulls^a^	STEX 185 °C, 7 min, 1% (w/w) H_2_SO_4_
Corn stover^a^	STEX 200 °C, 10 min, 0.2% (w/w) H_2_SO_4_
Sugarcane bagasse^a^	STEX 200 °C, 10 min, 0.2% (w/w) H_2_SO_4_
Wheat straw^a^	STEX 190 °C, 10 min, 0.2% (w/w) H_2_SO_4_
Spruce^b^	STEX 205 °C, 7 min, 1.5% (w/w) SO_2_
Softwood logging residues^b^	STEX 205 °C, 7 min, 1.5% (w/w) SO_2_
Birch^b^	STEX 200 °C, 5 min, 2.5% (w/w) SO_2_

^a^ Non-woody hydrolysates. ^b^ Woody hydrolysates

STEX, steam explosion

### Cultivation conditions

All cultivations were performed at 30 °C.

A BioLector I (M2p-labs GmbH) was used for high-throughput screening. For the pre-inoculum, 10 μL yeast cells were inoculated from a cryo-stock into 5 mL Delft medium in 50-mL tubes and grown overnight with shaking at 200 rpm. Overnight cultures were then inoculated at an optical density of 600 nm (OD_600_) = 0.4 into a final volume of 200 μL using CELLSTAR black clear-bottom 96-well microtiter plates (Greiner bio-one) and sealed with AeraSeal films (Sigma-Aldrich). The screening was performed in 85% humidity and shaking at 900 rpm for 36 h.

For flask cultivations, overnight cultures as described above were first re-inoculated into 25 mL Delft medium in 250-mL baffled flasks and grown shaking for another 24 h to increase the cell mass. For oxygen-limited flask screening, 20-mL cultures were inoculated at OD_600_ = 0.5 in 100-mL non-baffled flasks, which were then sealed with trap loops containing glycerol to create oxygen-limited conditions [21]. The shaker was set to 150 rpm (rotation radius of 12.5 mm). For measurements of lag phase and specific growth rate, growth was recorded using the Cell Growth Quantifier (Scientific Bioprocessing) by taking scattered light measurements every 10 min for 48 h. In addition, samples at the beginning (t0h) and at the end (t48h) of the screening were taken to determine cell dry weight, as well as glycerol and ethanol yields. Samples were first centrifuged at 3500 rpm for 5 min, after which the pellet was washed twice with water and dried in pre-weighted Eppendorf tubes for cell dry weight measurements, while the supernatant was filtered through 0.2-µm nylon membrane filters (VWR) for composition analysis. Yields were computed as the amount of glycerol or ethanol produced during the cultivation divided by the consumed hexose amount. Pentoses were not considered in yield computation as the selected strains cannot metabolise them. For aerobic flask screenings, 75 mL cultures at OD_600_ = 0.25 were inoculated in 500-mL baffled flasks. The shaker was set to 230 rpm (rotation radius of 20 mm). Samples were taken every 4 h during 24 h for OD_600_ and biosensor output measurements.

### Determination of substrate composition

Hydrolysate composition was determined by high-performance anion exchange chromatography with pulsed amperometric detection (HPAEC–PAD) for monosaccharides (glucose, galactose, mannose, xylose, and arabinose) and high-performance liquid chromatography (HPLC) for inhibitors (formic, acetic, and levulinic acids, furfural, and hydroxymethylfurfural) (Table 1). Samples collected during anaerobic flask screening were analysed by HPLC for glucose, ethanol, and glycerol and with HPAEC–PAD for galactose and mannose.

The HPLC system was equipped with a refractive index detector (Jasco) and a Rezex ROA–organic acid H^+^ column (Phenomenex). Separation was performed at 80 °C with a flow rate of 0.8 mL/min and 5 mM H_2_SO_4_ as eluent.

HPAEC–PAD was performed on an ICS5000 system equipped with a 4 × 250 mm Dionex Carbopac™ PA1 column and 4 × 50 mm guard column maintained at 25 °C (Dionex), with 10-μL injection volume. The eluents were: (A) water, (B) 300 mM NaOH, and (C) 100 mM NaOH + 85 mM sodium acetate. The samples were eluted isocratically with 100% eluent A for 20 min (1 mL/min) and detected with postcolumn addition of solvent B at 0.5 mL/min. Thereafter, a cleaning step with 20% eluent A, 40% eluent B, and 4% eluent C was performed at 1 mL/min for 11 min. Peak analysis was performed using Chromeleon software 7.2.10. Peaks were quantified against pure monosaccharide standards (0.1–0.005 mg/mL).

### Fluorescence analysis

Fluorescence analysis was carried out in a BioLector I as described previously [11]. The following filters were used: E-OP-301 (for biomass, gain 10), E-OP-315 (for ymYPET, gain 45), E-OP-309 (for mTurquoise2, gain 45), E-OP-319 (for mCherry, gain 55), E-OP-341 (for UV-GFP, gain 20), and E-OP-304 (for GFP, gain 40). Background fluorescence from the medium and the parental strains was subtracted and the signal normalised with either a normalisation construct or wavelength, as previously described [11]. Screening and analysis were carried out by treating triplicates individually, with the mean and standard deviation computed at the end.

For microscopy, 1-mL samples from aerobic flask screening were first washed in phosphate-buffered saline at pH 5 and centrifuged for 3 min at 3000 rpm. Cells, even those harvested at low OD_600_ values, were resuspended to obtain a dense solution, from which 1 μL was placed on a glass slide prior to visualisation. Samples were analysed on an inverted Leica DMI 4000 B fluorescence microscope (Leica Microsystems) equipped with a 100 × objective. The filters used were: excitation 441/30 nm and emission 480/80 nm (DC 455 nm) for mTurquoise2 (exposure 150 ns, gain 1.5); excitation 500/40 and emission 535/60 nm (DC 515 nm) for ymYPET (exposure 100 ns, gain 1); and excitation 546/24 and emission 605/150 nm (DC 560 nm) for mCherry (exposure 100 ns, gain 1). Gains and exposures were set prior to the experiment, so that none of the parental strains (without fluorescent proteins) would give a signal. Pictures were then analysed in Fiji by developing a macro for bulk analysis [22]. At least 25 cells/sample were used for fluorescence and robustness analysis.

### Robustness analysis

Robustness quantification was carried out using the following equation [2]:

1 $$R= -\frac{Fano\, factor}{mean} = -\frac{{\sigma }^{2}}{\overline{x} }*\frac{1}{m}$$

The ratio “σ^2/x” represents the Fano factor [4].

When computing robustness across conditions, R(c), “σ” and “x” refer to the standard deviation and mean, respectively, of a function, such as specific growth rate or lag phase, across multiple media (conditions) for one single strain (system). Instead, “m” refers to the mean of a function across all media in all strains. Therefore, R(c) identifies how stable a function is in the face of different conditions tested.

When computing robustness across systems, R(s), “σ” and “x” refer to the standard deviation and mean, respectively, of a function, such as specific growth rate or lag phase, across all systems (strains) for each condition (medium). Instead, “m” refers to the mean of a function across all strains and conditions. Therefore, R(s) identifies how similar a function is across different strains for each condition.

When computing robustness over time, R(t), “σ” and “x” refer to the standard deviation and mean, respectively, of a function (the biosensor signal output) throughout the screening for each replicate of each strain in each condition. Instead, “m” refers to the mean of a function across all strains. Therefore, R(t) identifies how stable a function is over time in each condition.

When computing robustness across populations, R(p), “σ” and “x” refer to the standard deviation and mean, respectively, of a function (the biosensor signal output) across all cells at each timepoint. Instead, “m” refers to the mean of a function across all strains, conditions, and timepoints. Therefore, R(p) identifies how stable a function is across a cell population (in other words, how homogeneous it is).

### Data analysis and availability.

Data analysis was carried out in R [23]. All data, R scripts, and the Fiji macro used in this study are available via GitHub (https://github.com/lucatorep/Robustness_implementation) or through the corresponding author.

## Results

### Experimental setup: perturbation space and strain selection

The perturbation space investigated in our study included seven different lignocellulosic hydrolysates obtained from non-woody (wheat straw—WSH, corn stover—CSH, oat hulls—OHH, and sugarcane bagasse—SBH) or woody (high-gravity spruce—HGSH, softwood logging residues—SLRH, and birch—BiH) plant biomass. The numerous inhibitory compounds and harsh conditions left after pre-treatment pose some of the challenges when using lignocellulosic hydrolysates as substrates for microbial fermentation [8]. For example, aldehydes and aromatic compounds cause redox imbalance and oxidative stress [24–26], weak acids cause acidification of the cytosol and metabolic stress [27, 28], and high sugar levels cause osmotic stress [29] as well as product inhibition upon their conversion into ethanol [30]. Owing to differences in plant biomass compositions, hydrolysates contain different types and amounts of inhibitory compounds (Table 1), with differential effects on cell performance (see, Additional file 1).

Among the *S. cerevisiae* strains employed in this study, CEN.PK113-7D is a laboratory strain originating from wild-type and industrial strains [31], whereas Ethanol Red and PE2 are widely used for first-generation bioethanol production in Europe and Brazil, respectively [32]. The performance of functions related to growth and intracellular parameters was here evaluated. Next, robustness was quantified to assess the stability of these functions across conditions, strains, time, and population.

### Ethanol Red outperforms the other strains in oxygen-limited flask screening

**Fig. 1:**
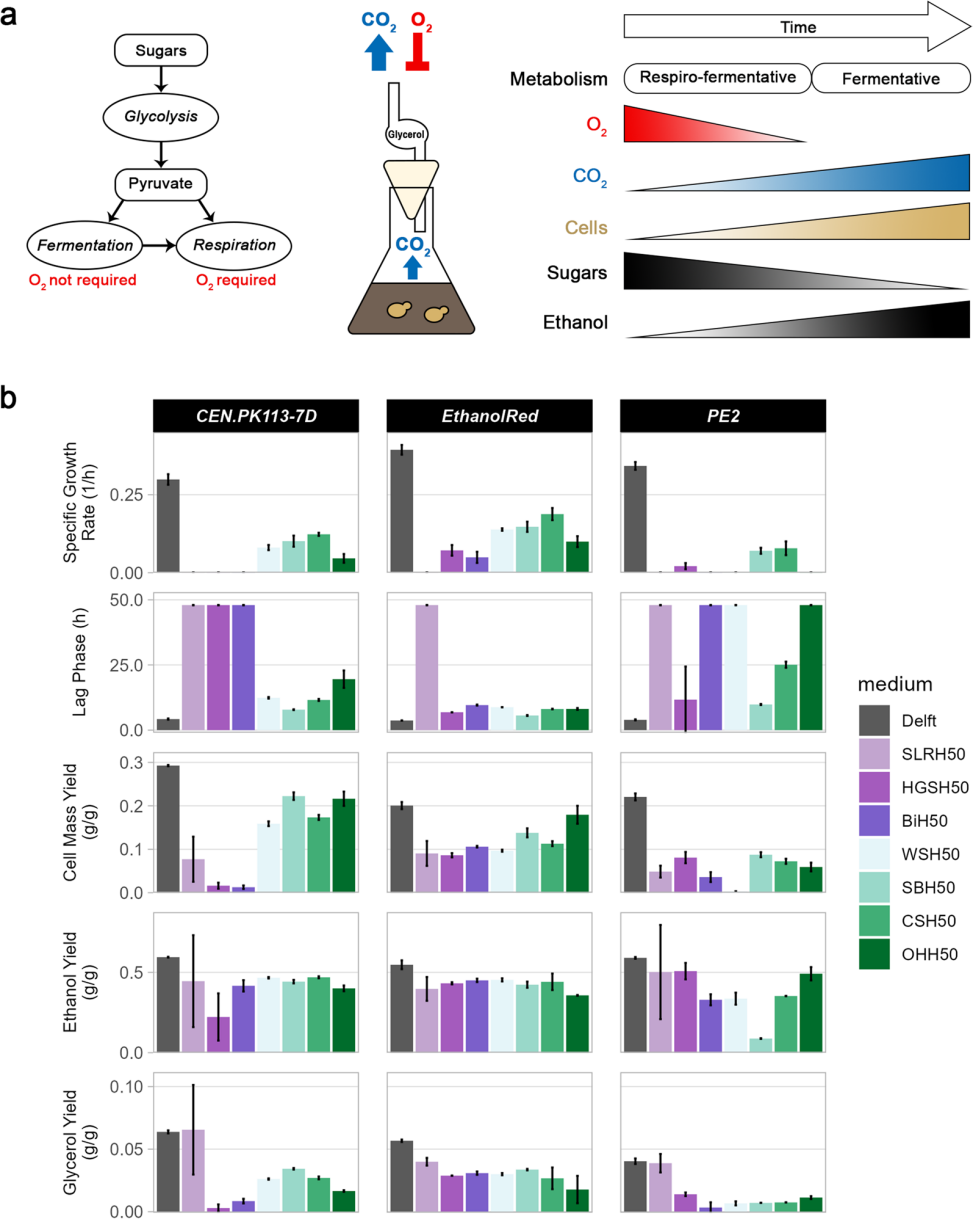
Overview of oxygen-limited screening and strain performance in various lignocellulosic hydrolysates. **a** Yeast can metabolise sugars through fermentation or respiration depending on oxygen and substrate availability. Using a glycerol trap loop to close the cultivation flask blocked the diffusion of oxygen from outside, causing the cells to rely exclusively on the oxygen trapped inside. Hence, yeast employed a respiro-fermentative metabolism until oxygen was available, but switched to a fermentative metabolism thereafter. **b** Overview of five functions (specific growth rate, lag phase, ethanol/glycerol/cell mass yields) of three S. cerevisiae strains (CEN.PK113-7D, Ethanol Red, PE2) in seven different lignocellulosic hydrolysates at 50% (vol/vol) and a control condition (Delft). Shades of green refer to non-woody hydrolysates, while shades of purple refer to woody hydrolysates

In 2G biofuel production, sugars present in the lignocellulosic hydrolysates are converted to ethanol. Because of the Crabtree effect, *S. cerevisiae* can exhibit fermentative (absence of oxygen and abundance of sugars), respiro-fermentative (presence of oxygen and abundance of sugars) or respiratory (presence of oxygen and low levels of sugars) growth [33]. Therefore, to avoid ethanol re-consumption while still allowing the oxygen-required biosynthesis of sterols and unsaturated fatty acids necessary for microbial growth [34, 35], yeast strains were grown in media containing 50% (vol/vol) lignocellulosic hydrolysates under oxygen-limited conditions (Fig. 1a). In this setup, the only oxygen available for the cells was the one inside the flask at the beginning of the screening, as the glycerol trap loop prevented diffusion of oxygen from outside. Ethanol Red grew in almost all hydrolysates, whereas PE2 and CEN.PK113-7D did not (Additional file 1: Fig. S1a). Moreover, Ethanol Red displayed the highest ethanol and glycerol yields and specific growth rate, along with the shortest lag phase (Fig. 1b). In contrast, despite its scarce growth, PE2 attained relatively high cell mass and ethanol yields, but low glycerol yields (Fig. 1b). Sterols and unsaturated fatty acids are essential for microbial growth and play a key role in cell tolerance towards many forms of stress [36–38]. However, their biosynthetic process is energy-consuming as one molecule of ergosterol requires 24 molecules of ATP and 16 of NADPH + H^+^ [35]. Variations in strain performance in the hydrolysates tested might be due to strain-specific differences in lipid and sterol composition of membranes, as well as their metabolic activities. Ergosterol has been shown to act as a cell protectant against inhibitory compounds [39]. Therefore, we speculate that when cultivated in lignocellulosic hydrolysates, Ethanol Red might rely on a basal metabolic state which allows it to divert energy towards growth rather than tolerance.

### Robustness across conditions unveils strains with the most stable performance across hydrolysates

**Fig. 2:**
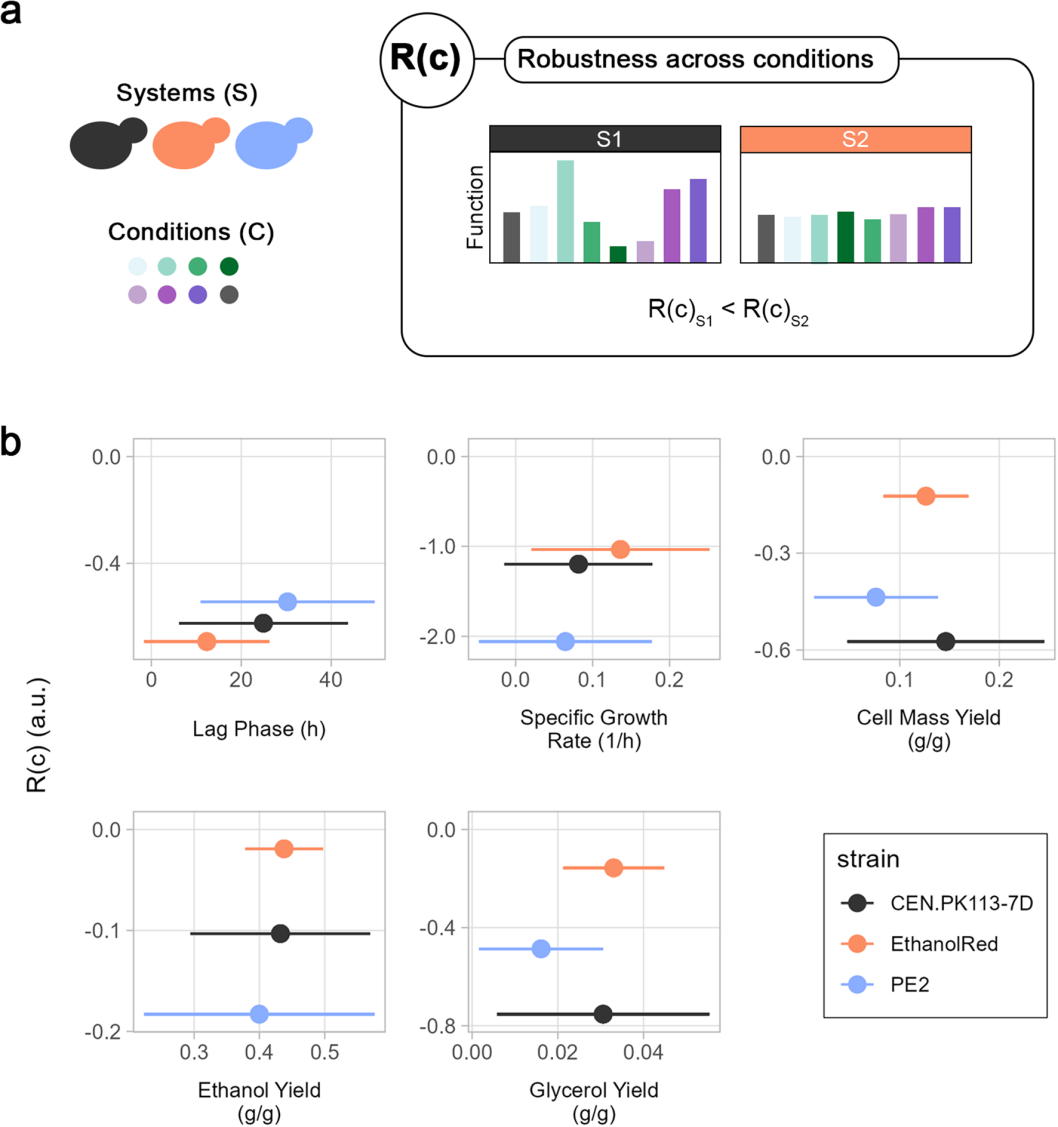
Robustness over perturbation in oxygen-limited flask screening. **a** R(c) denotes how stable a function of a system (S) is in the face of different conditions (C). In the example, the function is more robust in system 2 than in system 1, because it is more stable across all conditions. **b** Correlation between performance and robustness for five functions. Dispersion of data on the *x*-axis refers to the standard deviation of performance across all conditions (lignocellulosic hydrolysates)

During industrial bioprocesses, cell factories’ performance and productivity are constantly challenged by multiple perturbations and varying conditions [1]. Here, the robustness formula (Eq. 1) and the data collected from oxygen-limited flask screening were used to assess the robustness across conditions (different hydrolysates), R(c) (Fig. 2a). Ethanol Red exhibited both the best performance—short lag phase, highest specific growth rate, and ethanol and glycerol yields—as well as the highest robustness for multiple functions (Fig. 2b). This finding confirmed the elevated tolerance and robust functions associated with Ethanol Red [2, 3, 40]. Among all the functions considered, ethanol yield achieved the highest R(c) values. In the absence of oxygen, fermentation is the only source of energy for ATP production, and ethanol is the final product. Therefore, maintaining a stable (robust) ethanol yield is necessary for cells to cope with the stressful environment of lignocellulosic hydrolysates.

Based on the definition of robustness, it is not possible to make assumptions about performance from the robustness value alone. In fact, owing to trade-offs, the best-performing strain might not necessarily be the one with the highest robustness for a certain function [2, 3]. For example, CENPK113-7D showed the highest robustness values for specific growth rate and lag phase in woody substrates, even though it was the worst-performing strain (Additional file 1: Fig. S1b).

### Robustness over systems evaluates the impact of perturbations on strain performance

**Fig. 3:**
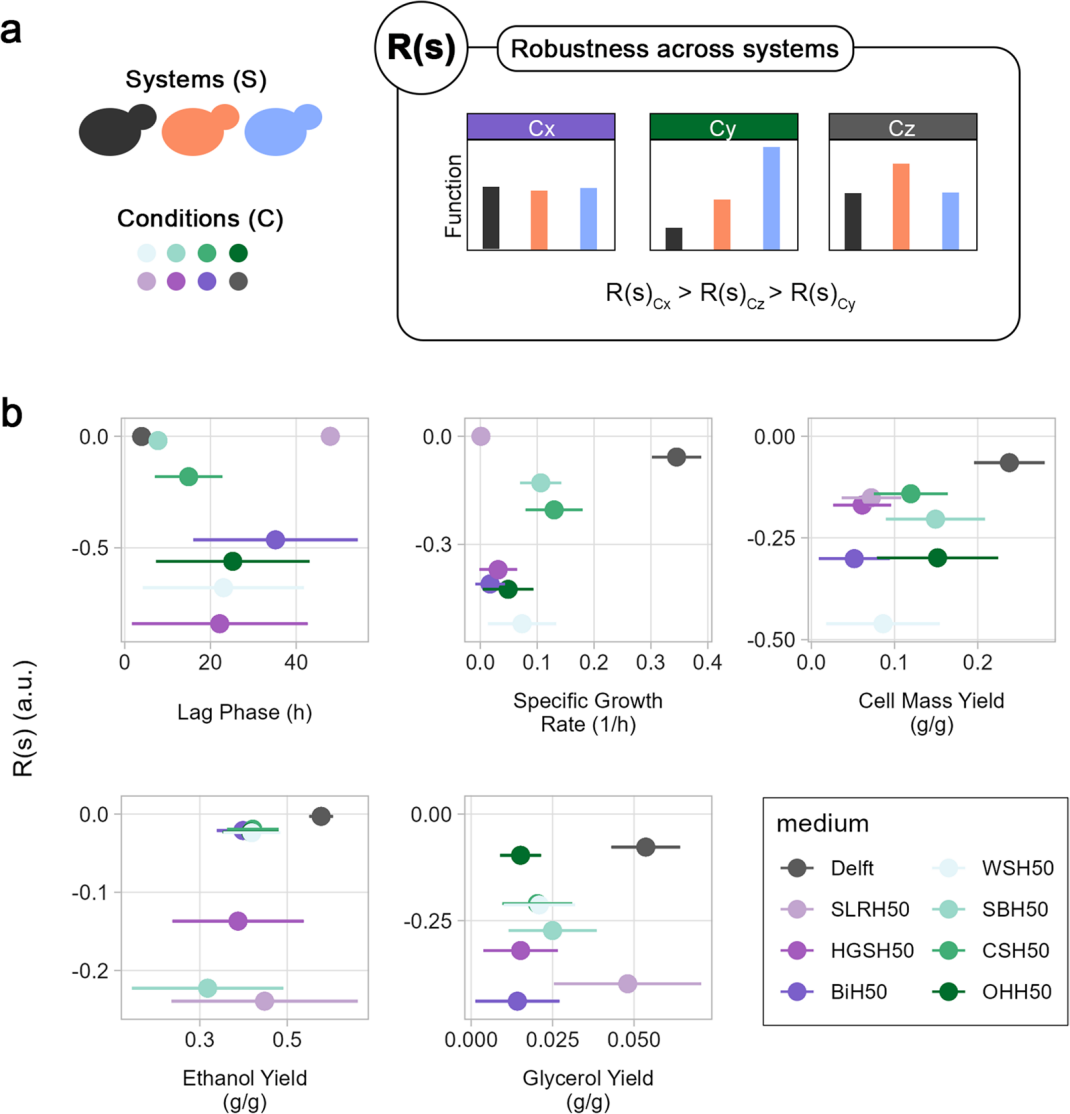
Robustness over systems in oxygen-limited flask screening. **a** R(s) denotes how stable a function is across systems (S) in the same condition (C). In the example, the most similar performance across systems is in perturbation x, followed by z and y. This identifies how similarly different systems react to the same perturbation. **b** Correlation between performance and R(s) of five functions. Dispersion of data on the *x*-axis refers to the standard deviation of performance among all strains

Substrate composition is one of the main determinants of microbial performance and physiological responses. Understanding differences and similarities in how strains respond to specific conditions can help direct strain engineering. Therefore, to assess the impact of the medium on cell metabolism across strains, the robustness formula (Eq. 1) was applied to compute robustness across systems, R(s). In this case, R(s) identified the similarity of a function across different yeast strains for each substrate (Fig. 3a).

Using the oxygen-limited flask screening data, R(s) pointed to how similarly the three strains were performing in each medium. The highest R(s) was observed in the control condition (Fig. 3b), which could be explained by the absence of stressors and, therefore, optimal and similar behaviour among strains. R(s) of lag phase and specific growth rate was high also for SLRH50, but that was because of a lack of growth in all the strains (Fig. 3b). Non-woody hydrolysates led to higher cell mass yields than woody ones, although no clear distinction could be observed with respect to the corresponding R(s). This suggests that non-woody hydrolysates exerted a smaller inhibitory effect on cell mass yield compared to woody hydrolysates (difference in performance) and not all strains responded equally (scattered R(s)). For glycerol yield, all media led to similar performance, thereby preventing any clustering based on the hydrolysate category. Nevertheless, woody hydrolysates emerged as having lower R(s) for glycerol yield than non-woody ones. Two hydrolysates stood out in this function: OHH50 and SLRH50. On one hand, Delft and OHH50 exhibited very similar R(s), but three times stronger performance in the former, suggesting that all strains were affected equally by OHH50. On the other hand, Delft and SLRH50 displayed very similar performance, but different R(s), suggesting that some strains improved the performance and others decreased it, which led to the same mean performance but lower R(s).

Computing R(s) using the experimental yields and rates is better suited when microorganisms belonging to the same species are compared, as they share the same maximum theoretical production rates or yields. However, when comparing microorganisms from different species or genera, this would make R(s) irrelevant. In this case, efficiency (ratio between the experimental and theoretical yields or rates) rather than rates/yields themselves can be used, thus allowing to compare relative changes in functions rather than absolute values. However, note that the function analysed in R(s) computation would be the efficiency, not the experimental rates or yields, thus making R(s) a measurement of the similarity of product rate or yield efficiency across strains for each condition.

### High-throughput investigation of eight intracellular parameters with biosensors

**Fig. 4:**
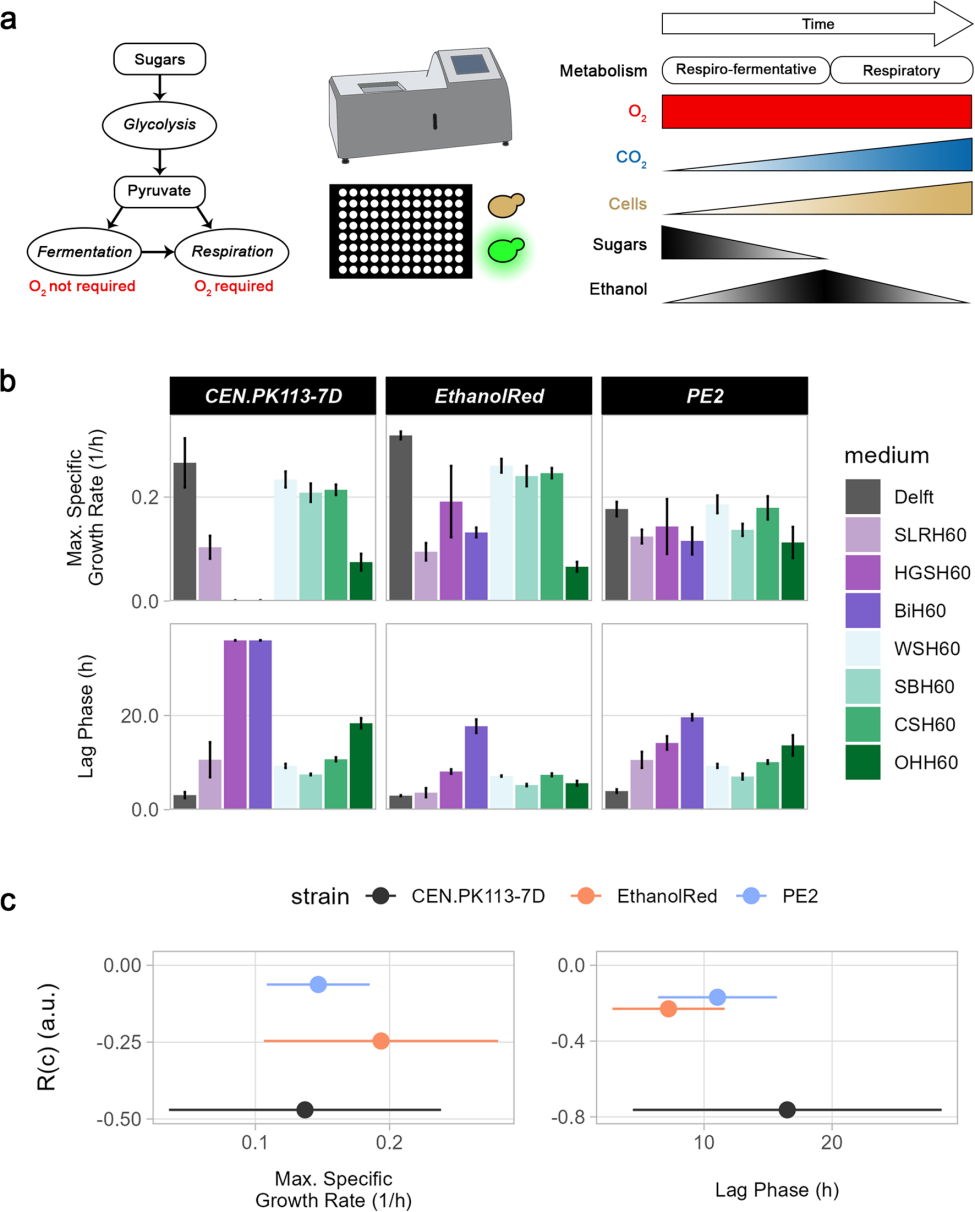
High-throughput aerobic screening overview, strain performance, and R(p) in various lignocellulosic hydrolysates. **a** Overview of the high-throughput aerobic screening, in which fluorescent biosensors were used to assess eight intracellular parameters. S. cerevisiae uses a respiro-fermentative metabolism until sugars are consumed and then switches to respiratory metabolism, whereby ethanol is consumed as a carbon source. **b** Performance of two functions (specific growth rate and lag phase) of three S. cerevisiae strains (CEN.PK113-7D, Ethanol Red, and PE2) in seven different lignocellulosic hydrolysates at 60% (vol/vol) and a control condition (Delft). Shades of green refer to non-woody hydrolysates, while shades of purple refer to woody hydrolysates. **c** Correlation between performance and R(c) of specific growth rate and lag phase. Dispersion of data on the *x*-axis refers to the standard deviation of performance across all conditions (media)

Real-time monitoring of the intracellular environment in a bioprocess is limited by a lack of suitable tools. Fluorescent biosensors can be employed to overcome this limitation [11], but oxygen needs to be present in the cultivation to allow proper formation of the chromophore [41]. Therefore, the three biosensor-bearing strains were tested under aerobic conditions in a high-throughput system in the selected perturbation space, with the aim of following eight intracellular parameters over time (Fig. 4a). In line with previous findings, no differences in lag phase and maximum specific growth rate were detected between the parental strains and the strains bearing biosensors (Additional file 1: Fig. S2), suggesting no impact of the biosensors on yeast metabolism [11, 12]. In contrast to oxygen-limited conditions in flask screening, nearly all strains grew in media containing 60% (vol/vol) lignocellulosic hydrolysate (Additional file 1: Fig. S3a). Ethanol Red remained the best-performing strain, with overall higher specific growth rates and shorter lag phases (Fig. 4b). However, PE2 exhibited the highest R(c) for both functions across all media (Fig. 4c) or when categorising them into woody and non-woody (Additional file 1: Fig. S3b). With oxygen present in the medium, carbon flux could be channelled to respiration instead of fermentation, thereby increasing energy production and the chances of surviving the harsh conditions in lignocellulosic hydrolysates. Moreover, the good performance of Ethanol Red might be due to its increased biosynthesis of ergosterol, with its cell-protecting properties, under aerobic conditions when exposed to lignocellulosic inhibitors [42].

**Fig. 5:**
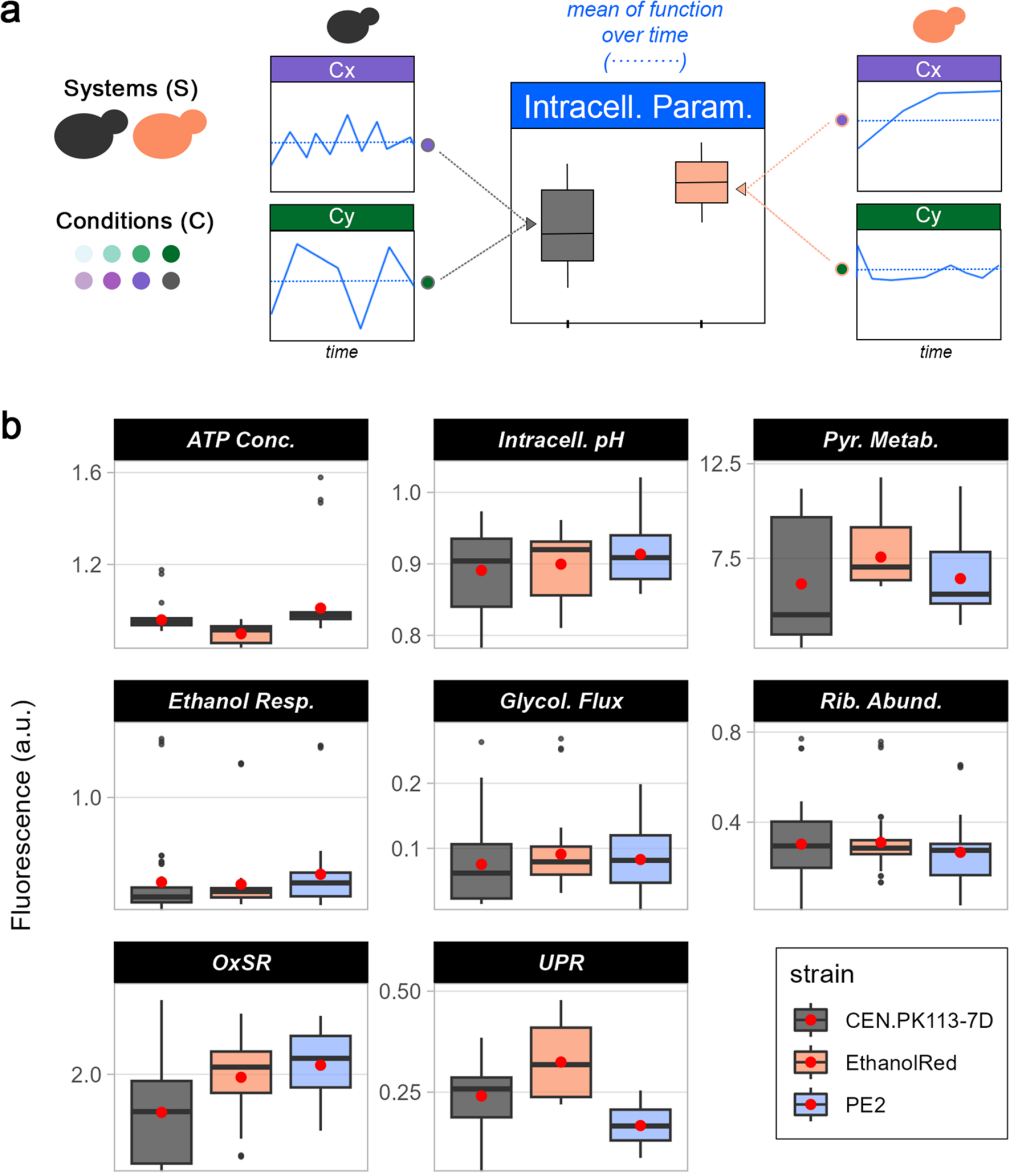
Overview of intracellular parameters in high-throughput aerobic screening. **a** For each biosensor (intracellular parameter), strain (system), medium (condition), and replicate, the mean fluorescence output over the time course of the screening (blue dotted line) was computed. Each mean was then used to construct the boxplots for each strain. **b** Overview of biosensor output for eight intracellular parameters (left to right, top to bottom): ATP level, intracellular ATP, pyruvate metabolism, ethanol respiration, glycolytic flux, ribosome abundance, oxidative stress, and unfolded protein response. Red dots identify the mean across all media and replicates in each strain

Over the course of 36 h, eight intracellular parameters, including ATP concentration, intracellular pH, pyruvate metabolism, ethanol consumption, glycolytic flux, ribosome abundance, oxidative stress response (OxSR), and unfolded protein response (UPR), were monitored (Additional file 1: Fig. S4). The mean fluorescent output for each strain, replicate and medium across the 36 h was then computed to quantify the intracellular parameters (Fig. 5a). Ethanol Red displayed higher pyruvate metabolism and UPR with respect to the other strains, whereas PE2 presented higher ATP, intracellular pH, ethanol respiration, and OxSR (Fig. 5b). Glycolytic flux and ribosome levels were comparable between the three strains. Differences in stress responses were detected according to the origin of plant biomass (Additional file 1: Fig. S5). While OxSR was higher in non-woody hydrolysates, UPR was higher in woody ones, especially during the exponential phase. This was in line with expectations based on hydrolysate composition (Table 1). Woody hydrolysates are richer in weak acids, which mainly elicited UPR [43, 44], whereas non-woody hydrolysates present more aldehydes, causing oxidative stress [26]. When the biosensor output was analysed according to growth phase, pyruvate metabolism was higher during exponential phase than lag phase, pointing to higher fermentation activity. However, no major differences in glycolytic flux were seen. This suggested that the sugars were directed towards a fermentative or respiratory pathway during the two phases. Respiration has been shown to be a key determinant of the length of lag phase, with cells capable of a respiratory metabolism growing earlier than fermenting ones [45]. Ethanol consumption was instead at its lowest in exponential phase, with few differences among strains and media (Additional file 1: Fig. S5). The biosensor output for ethanol consumption decreased in exponential phase due to glucose repression of the fluorescent protein’s promoter [46]. Intracellular pH and ATP levels were instead stable in both lag and exponential phase, as the abundance of hexoses stabilised ATP production and intracellular pH. In general, the latter was slightly lower in non-woody compared woody hydrolysates, which might be explained by abundant weak acids in the medium [28].

### Robustness over time establishes the degree of intracellular parameter fluctuations

**Fig. 6:**
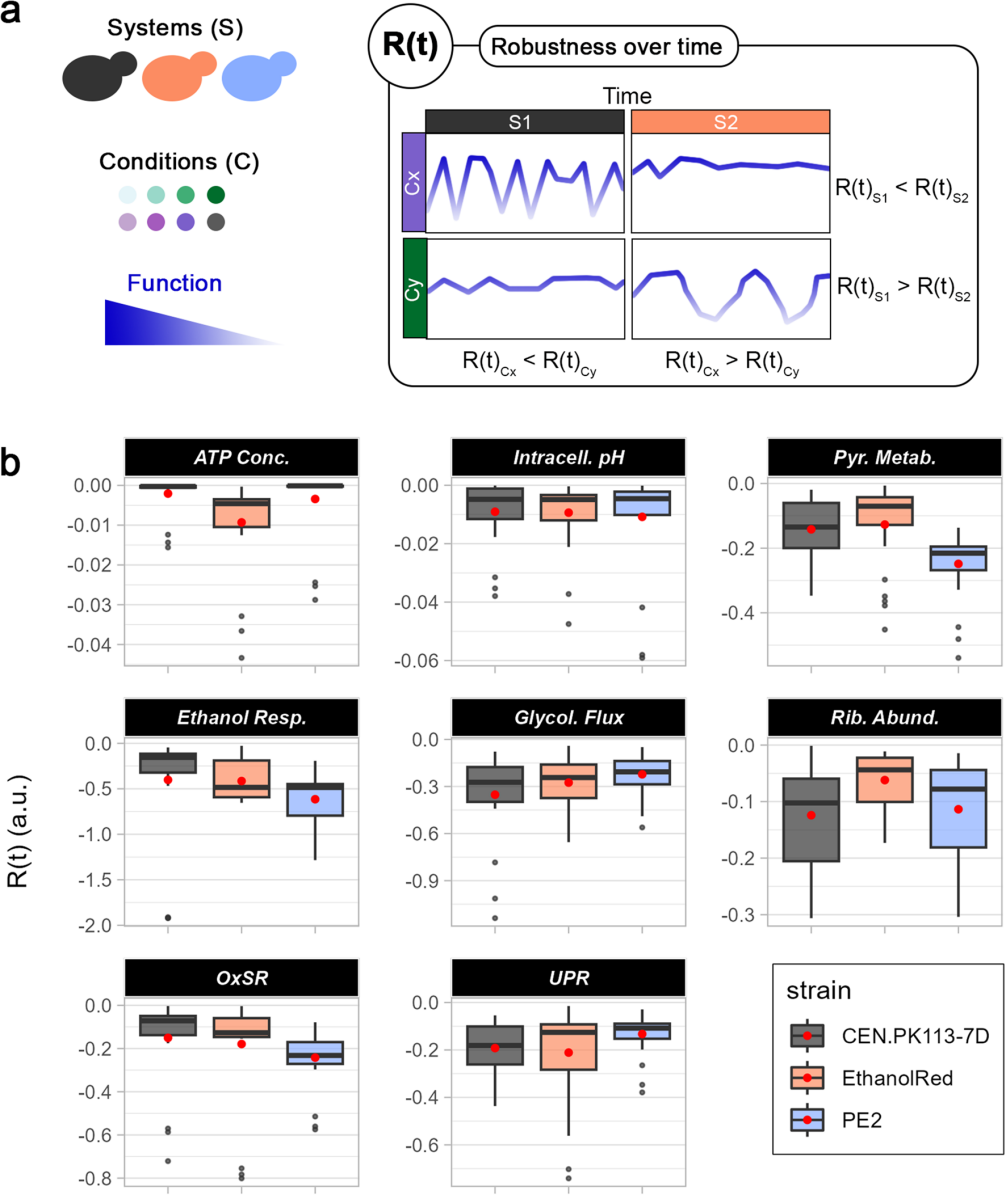
Robustness over time for intracellular parameters. **a** R(t) denotes how stable a function of systems (S) is over time for each condition (C). R(t) can be used to compare the same perturbation between different strains (systems) or within the same strain to assess how different media affect function stability. **b** R(t) for eight intracellular parameters (left to right, top to bottom): ATP level, intracellular ATP, pyruvate metabolism, ethanol respiration, glycolytic flux, ribosome abundance, oxidative stress, and unfolded protein response. Red dots identify the mean R(t) across all media and replicates for each strain

Fluorescent biosensors allow for monitoring trends in the intracellular environment. Studying the stability of a function over time will provide an important understanding of yeast metabolism. Such information would be of importance for determining the suitability for industrial purposes. For example, the stability of ATP levels correlates with cytotoxic protein aggregation [47], whereas, by looking into production rates over time of different strains, one can assess which strain has the most stable production. Therefore, a quantitative tool for assessing the stability of functions over time would facilitate such analyses. The robustness quantification formula (Eq. 1) was employed here to evaluate robustness over time, R(t). Higher R(t) values were associated with more stable functions and, hence, the ones with the less data dispersion over time with respect to their mean (Fig. 6a). Applying the formula to assess the stability of biosensor fluorescent output (Fig. 6b) revealed that CEN.PK113-7D attained the most stable ATP level, ethanol consumption, and oxidative stress over time. In contrast, PE2 exhibited the highest R(t) for UPR and glycolytic flux, whereas Ethanol Red had the highest R(t) for pyruvate metabolism and ribosome levels (Fig. 6b). In the case of OxSR and UPR, low R(t) indicated greater activation of the stress response itself. In fact, the elevated UPR and OxSR in Ethanol Red and PE2, respectively (Fig. 5b), are associated with low R(t) (Fig. 6b). Stable ATP level throughout the cultivation is a desired trait, as it would suggest enough energy to sustain all the metabolic processes. On the other hand, being able to switch on/off specific metabolic responses is crucial for survival under stressful conditions to maximise energy utilisation. Therefore, high R(t) is not necessarily a desired trait, but it depends on the function considered. PE2 displayed the least stable pyruvate metabolism and ethanol consumption over time, suggesting it was the most flexible when switching between fermentation and respiration (Additional file 1: Fig. S4).

Except for intracellular pH and ethanol consumption, R(t) was generally higher in exponential phase than in lag phase, with clear relative differences among strains and media (Additional file 1: Fig. S6). The lag phase represents the time cells spend adapting to a new environment before they start growing exponentially [45]. This implies several metabolic rearrangements and, consequently, less stable intracellular parameters. Finally, woody hydrolysates showed overall lower R(t) compared to non-woody ones (Additional file 1: Fig. S6). This can be explained by their higher amounts of inhibitors, which result in greater growth variability, lower specific growth rates, and a more challenging environment for yeast strains to adapt to.

### Single-cell investigation of intracellular parameters highlights the presence of subpopulations

**Fig. 7:**
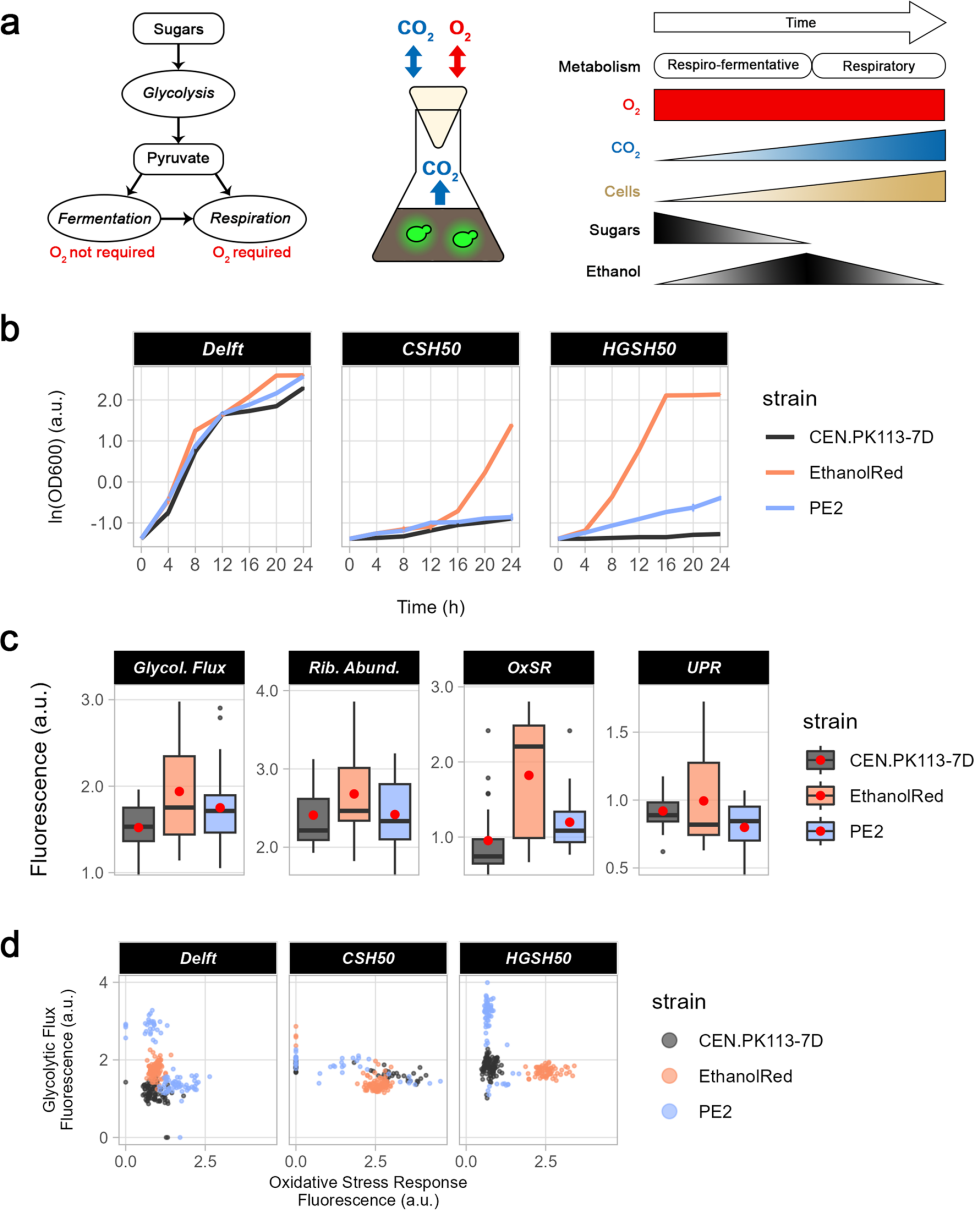
Growth, intracellular parameters, and subpopulations in aerobic flask screening. **a** Overview of the aerobic flask screening, in which fluorescent biosensors integrated into the genome of the three yeast strains were used to assess four intracellular parameters. S. cerevisiae presented a respiro-fermentative metabolism until sugars were abundant and then switched to a respiratory metabolism, whereby ethanol was consumed as a carbon source. **b** Growth curves of the three strains selected in lignocellulosic hydrolysates during aerobic flask screening. **c** Overview of biosensor output for four intracellular parameters (left to right): glycolytic flux, ribosome abundance, oxidative stress, and unfolded protein response. Boxplots were made by plotting the mean across all cells at each timepoint for all media. Red dots identify the mean across all media and timepoints in each strain. **d** Dot plot correlating oxidative stress and glycolytic flux at 20 h from the beginning of the screening for the three strains. Each dot represents a single cell

During cell cultivation, different isogenic subpopulations (i.e., subpopulations with the same genetic background) tend to form [13]. These subpopulations often show different physiological traits, such as different stress responses, growth, and/or productivity [48–50]. Understanding how subpopulations emerge is crucial for optimising protocols and tailoring yeast strains for targeted industrial purposes, ultimately enhancing yields and efficiency. Therefore, biosensors from the *Sc*EnSor Kit [12] were used to operate a single-cell investigation of intracellular parameters during yeast growth in lignocellulosic hydrolysates. Strains bearing either GlyOx (simultaneous detection of glycolytic flux and oxidative stress) or RibUPR (ribosome abundance and UPR) biosensors were grown in aerobic flasks in Delft, CSH50 or HGSH50 medium for 24 h (Fig. 7a). To assess whether subpopulations formed after inoculation, a low starting OD_600_ (0.25) was used. This caused CEN.PK113-7D and PE2 to have a very long lag phase in HGSH50 and CSH50, achieving only one doubling during the whole screening (Fig. 7b). Instead, Ethanol Red was able to grow in all lignocellulosic hydrolysates. Biosensor outputs were in line with the results obtained by high-throughput screening (Fig. 7c). Ethanol Red had the lowest glycolytic flux (low flux is associated with high fluorescence), followed by PE2 and CEN.PK113-7D (Fig. 7c); however, it was also the only strain in which glycolytic flux augmented over time (Additional file 1: Fig. S7a). Ethanol Red exhibited also the strongest stress responses (OxSR and UPR) and highest ribosome levels (Fig. 7c). Moreover, when looking at the distribution of biosensor output for each strain at each timepoint, PE2 and CEN.PK113-7D stood out as having the most diverse populations (Additional file 1: Figs. S7–S8).

To gain a more complete overview of yeast metabolism and its changes over time, the correlation between each intracellular parameter pair (measured by the GlyOx and RibUPR biosensors) was assessed for each strain and timepoint (Supplementary Figs. S9–S10, Additional File 1). Two subpopulations were noted for PE2 in Delft and HGSH50 media throughout the screening: one with high OxSR and glycolytic flux, and the other with low OxSR and glycolytic flux (Fig. 7d, Additional file 1: Fig. S9). Here, high glycolytic flux probably allowed cells to be more metabolically active and generate more ATP and NAD(P)H + H^+^ to sustain both growth and a strong OxSR. Alternatively, as this behaviour was observed in yeast grown in Delft and HGSH50 (with no or few compounds triggering oxidative stress, respectively), the higher glycolytic flux might correlate with higher respiro-fermentative metabolism. In turn, the higher respiration might increase the production of reactive oxygen species from basal metabolic processes, in turn requiring a more active OxSR. Both PE2 and CEN.PK113-7D harboured two subpopulations with equal glycolytic fluxes, but different OxSR levels in CSH50 (Additional file 1: Fig. S9). As CSH contained aldehydes, it might be that while one subpopulation was using the energy to actively detoxify the medium, the other was using it to grow. CEN.PK113-7D had also two subpopulations emerging over time; in one case they presented the same UPR but different ribosome abundance in Delft medium, while in the other, they shared the same ribosome levels but different UPR in HGSH50 (Additional file 1: Fig. S10). While differences in ribosomal proteins have been associated with varying cell lifespans [51], variations in UPR have not been reported before. PE2 showed an increasing UPR with similar ribosome abundance, but no clear distinction in subpopulations. Finally, no subpopulations of Ethanol Red appeared in any of the media tested for any of the two intracellular parameter pairs.

### Robustness across populations indirectly quantifies population heterogeneity

**Fig. 8:**
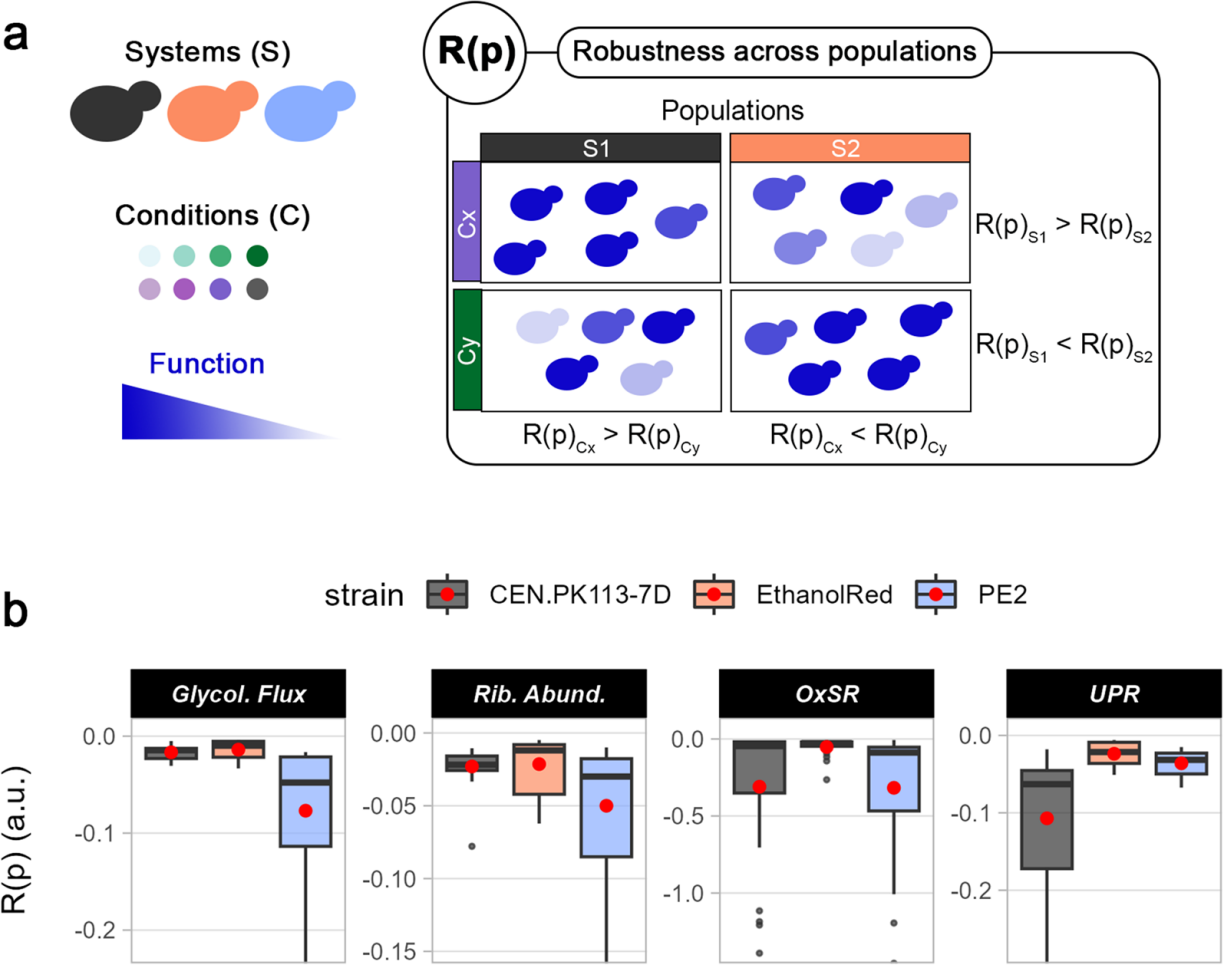
Robustness across populations to assess levels of population heterogeneity. **a** R(p) denotes how homogeneous (stable) a function of systems (S) is within cells in a population for each timepoint in each condition (C). This value can be used to compare the same condition between different strains (systems) or within the same strain to assess how different media affect function stability. **b** R(p) for four intracellular parameters (left to right): glycolytic flux, ribosome abundance, oxidative stress response, unfolded protein response. Boxplots include R(p) values for each timepoint in each medium tested. Red dots identify the mean R(p) across all media and timepoints for each strain

Phenotypic population heterogeneity refers to the rise of subpopulations with phenotypic differences from the same isogenic bulk population and it might hinder productivity in bioprocesses [1, 13]. For example, an early study showed that during L-valine production, only a minor part of the bulk population is actively generating L-valine, while the majority is producing biomass [52]. In insulin-producing yeast, three different subpopulations have been found under glucose-limited conditions, probably to increase fitness [50]. This phenomenon of distributing different phenotypes within a population is often referred to as bet-hedging, aimed to increase survivability in face of unexpected events [53]. Therefore, whether phenotypic population heterogeneity is a positive or negative trait depends on the function considered and the scenario. Here, using the robustness quantification formula (Eq. 1), we propose an efficient, quick, and easy way of indirectly quantifying population heterogeneity by computing robustness across populations, R(p) (Fig. 8a). R(p) gives an indication of how homogeneous a function is within a cell population at a specific timepoint. Therefore, the lower R(p), the higher the level of population heterogeneity for a function within a cell population. Using single-cell data obtained via aerobic flask screening, R(p) was computed for glycolytic flux, OxSR, UPR, and ribosome abundance (Fig. 8b). Ethanol Red exhibited the highest R(p) for all functions, confirming the low level of heterogeneity across all conditions observed with the scatter plots (Additional file 1: Figs. S9b–10b). PE2 displayed the highest degree of population heterogeneity for all functions except UPR, for which CEN.PK113-7D was the most heterogeneous (Fig. 8b). The above trend was valid also when media were considered individually (Additional file 1: Fig. S11). Similar to the other R types, also R(p) does not give any information on the performance by itself, thus cases with heterogeneous populations might still have an average performance higher than the one from homogeneous populations.

## Discussion

In the present study, three *S. cerevisiae* strains were cultivated in seven different lignocellulosic hydrolysates obtained from both woody- and non-woody plant biomass. Using a recently developed robustness quantification method [2], we here expanded the robustness concept by illustrating four different ways of implementing robustness analysis into canonical experimental procedures. Our approach allows the investigation of robustness (i.e., the ability of a system to maintain a stable performance) in quantitative terms, addressing different perspectives. Such tools are instrumental in moving strain and bioprocess development forward. The physiological characterisation was carried out by checking both growth-related functions (specific growth rate, ethanol yield, etc.) and the intracellular environment using fluorescent biosensors from the *Sc*EnSor kit [12]. Notably, the same concept can be applied to evaluate the robustness of titres, rates, and yields (TRY metrics) for an industrial process. A wide range of biosensors is available to detect, quantify, and monitor industrially relevant compounds, such as branched-chain amino acids [54], natural products [55], and short- or medium-chain fatty acids [56]. By coupling these tools and the robustness quantification method, one can assess the TRY metrics and their robustness while developing and/or improving strains for industrial purposes. Often, microorganisms exploited in bioproduction are selected based on performance. However, this might be generally compensated from a metabolic point of view by low TRY stability (i.e., lower robustness) due to trade-offs [2, 3]. Metabolic operations are constrained thermodynamically by an upper limit on Gibbs energy dissipation [57]. Similarly, cells must constantly decide whether they should allocate their energy towards the performance of specific functions in favour of their robustness or vice versa. Therefore, from an industrial point of view, having a lower-performing and more-stable production phenotype over time or across conditions might sometimes be preferred over a better-performing but unstable one, especially in the case of continuous cultivations.

**Fig. 9:**
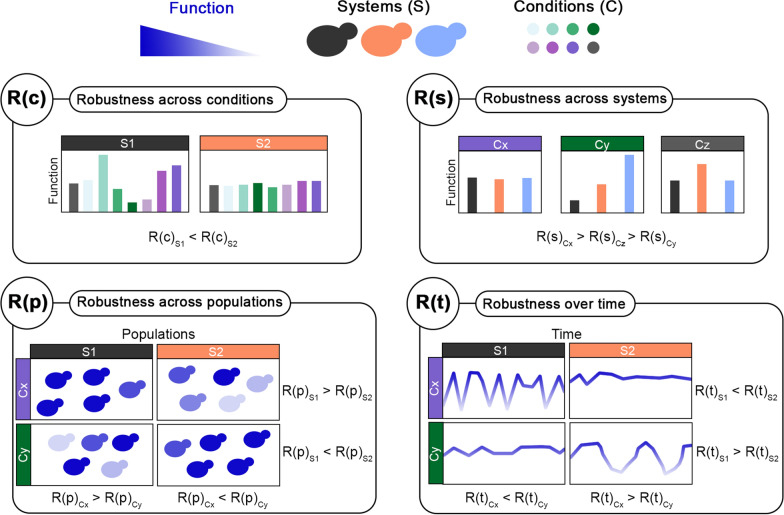
Summary of applications of the robustness quantification method for strain characterisation in this study. For a desired function (specific growth rate, ATP level, ethanol yield, cell size, etc.), systems (S), and set of conditions (C), it is possible to use the robustness quantification method to measure robustness across conditions (i.e., stability of a function across different conditions), systems (i.e., stability of a function across different systems), populations (i.e., stability of a function within a cell population), and over time (i.e., the stability of a function over time)

For the first time, four different applications of the same robustness quantification formula were here showcased (Eq. 1), each providing insights on different aspects of function stability (Fig. 9). First, R(c) was used to identify stable functions when the yeast strains were challenged with different conditions (different types of lignocellulosic hydrolysates in this case). Industrial bioprocesses encompass a wide range of conditions and perturbations that might affect microbial performance and productivity [1]. Therefore, following the selection of relevant conditions for a specific industrial process of interest, strains can be screened to evaluate their ability to withstand such challenges. R(s) was used to assess how similarly different strains responded to the same perturbation. This becomes useful in the early stages of strain development or characterisation of new strains/species, to understand how different perturbations affect microbial metabolism and performance. Next, R(p) offered a way to indirectly quantify population heterogeneity, as it measured how homogeneous a function was in a cell population. While this value can be used to identify the strain with the highest population heterogeneity for a function, it can be used also to assess which conditions increase such heterogeneity. Phenotypic population heterogeneity is a trait that might be positive or negative based on the function and scenario considered. When considering the production phenotype, generally a homogeneous population is preferred, so that every cell contributes to the production. While from a physiological point of view, in the case of bet-hedging, phenotypic population heterogeneity might instead be considered a positive feature. Similar considerations can be made also for R(t), which refers to the stability of a function over time. In fact, it might be more favourable to select a strain, whose production rate is not the highest possible, but is stable (robust) over time owing, maybe, to low heterogeneity. Such a strain may also give the highest productivity if one considers the overall process.

In the present work, we applied the robustness quantification method in a high-throughput screening system, flasks, and microscopy analysis to validate it at very different resolution levels: population, subpopulation, and single-cell. However, the same procedure can be extrapolated to analyse data from almost any experimental setup (e.g., large-scale reactors, gel-encapsulated cultures, or microfluidics chips) or instrument (e.g., flow cytometry to analyse thousands of cells simultaneously). Investigation of robustness and performance can be especially interesting in dynamic microfluidics single-cell cultivation, as it monitors growth, intracellular parameters, and production (via biosensors) of single cells over time while trying to mimic dynamic industrial conditions [58, 59].

Overall, the robustness quantification formula (Eq. 1) is a versatile equation that can be applied to many different scenarios consisting of at least two systems, a set of conditions (the more the better for statistical purposes), and function(s) of interest. As such, the use of the formula can be extended out of strain characterisation. For example, one can assess: (i) the stability of hydrolytic activity by an enzyme and its mutants across a set of temperatures or pH values; (ii) the stability of sugar release during pre-treatment of lignocellulosic biomass across time and different substrates; and (iii) specificity of a transcription factor in targeting a DNA consensus sequence. Applications are endless and only creativity becomes a bottleneck in finding new uses. It is, however, important to remember that robustness values computed with this formula are always relative (never absolute) and vary based on the selected perturbation space and strain.

## Conclusions

In the present work, we showcased for the first time four simple ways of implementing robustness quantification for the physiological characterisation of three *S. cerevisiae* strains cultivated in seven lignocellulosic hydrolysates. The physiological characterisation was carried out including the analysis of both growth-related functions (specific growth rate, ethanol yield, etc.) and eight intracellular parameters using fluorescent biosensors. The robustness quantification method was used to assess the stability of functions across (i) conditions (the seven hydrolysates), (ii) systems (three yeast strains), (iii) time, and (iv) cell populations. Moreover, the combination of different commonly available instrumentation validated the methods at resolution levels spanning from populations, through subpopulations and down to single cells. Owing to the simplicity of the robustness equation, it can be applied to many different scenarios having at least two systems to compare, a set of conditions, and function(s) of interest. In this way, robustness analysis offers a versatile tool for strain characterisation in multiple fields and, overall, for biotechnological applications.

### Supplementary Information

**Additional file 1: Table S1.** Trace metals and vitamin solutions. Composition of (A) trace metal and (B) vitamin solutions used in Delft medium. **Figure S1**. Growth curves and robustness across conditions in oxygen-limited flask cultivations. Overview of selected *S. cerevisiae* strains in flask screening under oxygen-limited conditions using different lignocellulosic hydrolysates. (a) Growth curves. (b) Correlation between performance and robustness across conditions, R(c), for five functions (lag phase, specific growth rate, ethanol/glycerol/cell mass yields) based on non-woody (WSH50, SBH50, CSH50, and OHH50) or woody (SLRH50, HGSH50, and BiH50) biomass. **Figure S2**. Maximum specific growth rates and lag phases. Maximum specific growth rates (a) and lag phases (b) of parental and biosensor strains in all tested media. Statistical differences between the biosensor and parental strains are represented above the bar plots; those between the control (Delft) and all other conditions are shown below the bar plots. As no differences in functions were observed between the parental and biosensor strains, all strains (three replicates each) have been used for a more reliable statistical analysis. *p ≤ 0.05; **p ≤ 0.01, ***p ≤ 0.001, and ****p ≤ 0.0001. **Figure S3**. Growth curves and robustness across conditions in BioLector screening. Overview of selected *S. cerevisiae* strains in flask screening under oxygen-limited conditions using lignocellulosic hydrolysates. (a) Growth curves. (b) Correlation between performance and robustness across conditions for two functions (lag phase and specific growth rate) dividing non-woody (WSH60, SBH60, CSH60, and OHH60) or woody (SLRH60, HGSH60, and BiH60) lignocellulosic hydrolysates. **Figure S4**. Line plots for intracellular parameters examined via BioLector I screening. Overview of all line plots for the biosensors used in BioLector screening. Cell mass is represented as the natural logarithm of scattered light, while intracellular parameters are denoted as fluorescence ratios (a.u.). **Figure S5**. Overview of biosensor outputs categorised by growth phase and medium. Overview of biosensor output for eight intracellular parameters computed either for the lag phase (left) or exponential phase (right) taking into consideration all tested media (“ALL”), only woody hydrolysates or only non-woody hydrolysates. Red dots identify the mean fluorescent output across all media in that group. Note that for glycolytic flux, the higher the biosensor output the lower the flux. **Figure S6**. Robustness over time of intracellular parameters categorised by growth phase and medium. Robustness over time (i.e., the stability of a function over time) for eight intracellular parameters computed either for the lag phase (left) or exponential phase (right). Robustness was computed taking into consideration all tested media (“ALL”), only woody hydrolysates or only non-woody hydrolysates. Red dots identify the mean R(t) across all media in that group. **Figure S7**. Line plots for GlyOx fluorescence outputs. Output from the GlyOx biosensor for glycolytic flux (a) and oxidative stress response (b). Violin plots represent the distribution of the intracellular parameter within the cell population at each timepoint. Red dots represent the mean across all cells. At least 25 cells were analysed for each timepoint. **Figure S8**. Line plots for RibUPR fluorescence outputs. Output from the RibUPR biosensor for ribosome abundance (a) and unfolded protein response (b). Violin plots represent the distribution of the intracellular parameter within the cell population at each timepoint. Red dots represent the mean across all cells. At least 25 cells were analysed for each timepoint. **Figure S9**. Scatter plots for GlyOx fluorescence outputs. Scatter plots summarising the correlation between glycolytic flux and oxidative stress response for each cell at each timepoint (0, 4, 8, 12, 16, 20, and 24 h) for CEN.PK113-7D (a), EthanolRed (b), and PE2 (c). Each dot represents a cell. At least 25 cells were analysed for each timepoint. **Figure S10**. Scatter plots for RibUPR fluorescence outputs. Scatter plots summarising the correlation between ribosome abundance and unfolded protein response for each cell at each timepoint (0, 4, 8, 12, 16, 20, and 24 h) for CEN.PK113-7D (a), EthanolRed (b), and PE2 (c). Each dot represents a cell. At least 25 cells were analysed for each timepoint. **Figure S11**. Robustness across populations for intracellular parameters categorised by medium. Robustness across populations (i.e., how homogeneous a function is within a cell population) for four intracellular parameters (top to bottom): glycolytic flux, ribosome abundance, oxidative stress response, and unfolded protein response. Red dots identify the mean R(p) across all timepoints for each strain in each medium.

## Data Availability

The data sets generated and/or analysed during the current study are available in the GitHub repository (https://github.com/lucatorep/Robustness_implementation) or from the corresponding author upon reasonable request.

## Abbreviations

2G: Second generation
WSH: Wheat straw hydrolysate
CSH: Corn stover hydrolysate
OHH: Oat hulls hydrolysate
SBH: Sugarcane bagasse hydrolysate
SLRH: Softwood logging residues hydrolysate
HGSH: High gravity spruce hydrolysate
BiH: Birch hydrolysate
R(c): Robustness across conditions
R(s): Robustness across systems
R(t): Robustness over time
R(p): Robustness across populations
OxSR: Oxidative stress response
UPR: Unfolded protein response
