# Supplementary material for: Four ways of implementing robustness quantification in strain characterisation
Source: Biotechnol Biofuels Bioprod. 2023 Dec 19;16:195. doi: 10.1186/s13068-023-02445-6 (PMC10729505; doi:10.1186/s13068-023-02445-6)
Supplement: Supplementary file 1 — Additional file 1: Table S1. Trace metals and vitamin solutions. Composition of (A) trace metal and (B) vitamin solutions used in Delft medium. Figure S1. Growth curves and robustness across conditions in oxygen-limited flask cultivations. Overview of selected S. cerevisiae strains in flask screening under oxygen-limited conditions using different lignocellulosic hydrolysates. (a) Growth curves. (b) Correlation between performance and robustness across conditions, R(c), for five functions (lag phase, specific growth rate, ethanol/glycerol/cell mass yields) based on non-woody (WSH50, SBH50, CSH50, and OHH50) or woody (SLRH50, HGSH50, and BiH50) biomass. Figure S2. Maximum specific growth rates and lag phases. Maximum specific growth rates (a) and lag phases (b) of parental and biosensor strains in all tested media. Statistical differences between the biosensor and parental strains are represented above the bar plots; those between the control (Delft) and all other conditions are shown below the bar plots. As no differences in functions were observed between the parental and biosensor strains, all strains (three replicates each) have been used for a more reliable statistical analysis. *p ≤ 0.05; **p ≤ 0.01, ***p ≤ 0.001, and ****p ≤ 0.0001. Figure S3. Growth curves and robustness across conditions in BioLector screening. Overview of selected S. cerevisiae strains in flask screening under oxygen-limited conditions using lignocellulosic hydrolysates. (a) Growth curves. (b) Correlation between performance and robustness across conditions for two functions (lag phase and specific growth rate) dividing non-woody (WSH60, SBH60, CSH60, and OHH60) or woody (SLRH60, HGSH60, and BiH60) lignocellulosic hydrolysates. Figure S4. Line plots for intracellular parameters examined via BioLector I screening. Overview of all line plots for the biosensors used in BioLector screening. Cell mass is represented as the natural logarithm of scattered light, while intracellular parameters are [file 13068_2023_2445_MOESM1_ESM.docx]

**Additional File 1**

**Four ways of implementing robustness quantification in strain characterisation**

**Luca Torello Pianale^1^, Fabio Caputo^1^, Lisbeth Olsson^1*^**

^1^Industrial Biotechnology Division, Department of Life Sciences, Chalmers University of Technology, Gothenburg, Sweden

***Correspondence:**

Prof. Lisbeth Olsson,

[lisbeth.olsson@chalmers.se](mailto:lisbeth.olsson@chalmers.se)

**SUPPLEMENTARY INFORMATION**

The “Supplementary Text” section includes an overview of lignocellulosic plant biomass composition and a detailed description of the inhibitors found in lignocellulosic hydrolysates.

The “Supplementary Tables” section includes a table with the trace metal and vitamin solution compositions used in this study (Supplementary Table S1).

The “Supplementary Figures” section includes:

- Growth curves and robustness across conditions in oxygen-limited flask cultivations (Supplementary Figure S1).
- Maximum specific growth rate and lag phase values for screenings in BioLector I, categorised by medium and strain (Supplementary Figure S2).
- Growth curves and robustness across conditions for screenings in BioLector I (Supplementary Figure S3).
- Line plots for intracellular parameters in BioLector screening (Supplementary Figure S4).
- An overview of biosensor outputs categorised by growth phase and medium (Supplementary Figure S5).
- Robustness over time of intracellular parameters categorised by growth phase and medium (Supplementary Figure S6).
- Line plots for GlyOx (Supplementary Figure S7) and RibUPR (Supplementary Figure S8) fluorescence outputs in aerobic flask screening.
- Scatter plots for GlyOx (Supplementary Figure S9) and RibUPR (Supplementary Figure S10) fluorescence outputs.
- Robustness across populations of intracellular parameters categorised by medium (Supplementary Figure S11).

**SUPPLEMENTARY TEXT**

**Overview of lignocellulosic plant biomass composition**

Lignocellulosic plant biomass is composed of 40%–50% cellulose, 25%–30% hemicellulose, and 15%–20% lignin, along with other extractives (1).

Cellulose is a glucose polymer abundant in plant cell walls, where it provides structure and mechanical strength (2).

Hemicellulose acts as a physical barrier for cellulose fibres, protecting and cross-linking them (3). Several hemicellulose types exist. Xyloglucan is the main hemicellulose in the primary cell wall of land plants (3,4). The hemicellulose type in the secondary cell wall varies highly with respect to plant species. Galactoglucomannan is the main hemicellulose component in the secondary cell wall of softwood trees, such as spruce or pine (5). Galactoglucomannan encompasses randomly distributed glucose and mannose residues polymerised in a backbone structure, with acetyl and galactosyl sidechains at position C2 and C3, and 1 acetyl group per 3–4 hexose units (6). Xylans are the main hemicellulose components in the secondary cell walls of hardwood trees (e.g., birch) and grasses (e.g. wheat, corn, sugarcane, and oat), and are composed mainly of pentose units (3). The substitutions on the xylan chain vary based on the type of plant, with glucuronoxylan found mostly in hardwood, arabinoxylan in non-woody materials (e.g., cereal grain and grasses), and glucuronoarabinoxylan in some agricultural crops and softwood (5%–10%) (3). In the xylan chain, there are generally 7 acetyl groups per 10 xylose units and 1 uronic acid residue for every 10 xylose residues (7). Moreover, in arabinoxylan and glucuronoarabinoxylan, the arabinose substitutions in the xylan backbone might be esterified with ferulic acid (8). Ferulic acids present in different chains are prone to cross-linking to form ferulic acid dimers. This renders the hemicellulose network stronger and less accessible to enzymes (8).

Lignin is a complex amorphous polymer formed by three phenyl-propane alcohols (p-coumarilic, coniferilic, and synapilic) as the main building blocks (9). Its main function is to provide hydrophobicity and structural rigidity, it is highly recalcitrant to enzymatic attack and, once degraded by pre-treatment, has a strong inhibitory effect on both enzymes and microbes (10).

**Formation of inhibitors in lignocellulosic hydrolysates and their effect on yeast metabolism**

The complex structure of lignocellulosic plant biomass hinders its degradation by microorganisms. Therefore, lignocellulosic plant biomass needs to be pre-treated to loosen its crystalline structure and improve access by hydrolysing enzymes and fermenting microorganisms (11). However, pre-treatment leads to the release of inhibitory compounds derived either from the plant biomass itself or its degradation products (12).

Structural polymers from cellulose and hemicellulose are degraded into aldehydes, such as furfural from pentoses and hydroxymethylfurfural from hexoses (12). These compounds are responsible for reduced biological activities, DNA damage, oxidative stress, and redox/energy imbalance. As ATP is used by ATP-binding-cassette transporters to pump the aldehydes out, NAD(P)H+H^+^ is used by enzymes to convert aldehydes into their respective alcohols, which exerts a lower inhibitory effect (13,14).

Weak acids in lignocellulosic hydrolysates originate either from further degradation of furfural and hydroxymethylfurfural (e.g., formic or levulinic acids) or are released from the hemicellulose fraction (e.g. acetic acid) (15). When the pH of the substrate is above the pKa of the weak acid (generally below 5), weak acids present in the medium in their protonated form can enter the cell via simple diffusion (16). Once inside at neutral pH, weak acids release a proton and remain trapped, lowering the intracellular pH (16). Therefore, yeast cells must use ATP to activate the H^+^-ATPase and other ATP-dependant transporters to pump the protons and acids out, causing ATP depletion and metabolic stress, which interferes with the glycolytic pathway (17,18).

Phenolic compounds are released from the lignin fraction as a mixture of aldehydes, ketones, acids, and alcohols, with varying degrees of growth inhibition (19). Overall, these compounds, especially aromatic ones, are very toxic at low concentrations. For example, ferulic acid is toxic at 0.2 g/L (20,21). Akin to aldehydes, phenolic compounds trigger oxidative stress and redox imbalance, as well as increased ergosterol production, mitochondrial activity, and membrane transports (21,22).

**SUPPLEMENTARY TABLES**

**Supplementary Table S1. Trace metals and vitamin solutions.** Composition of (A) trace metal and (B) vitamin solutions used in Delft medium.

| **A. Trace metal solution 1000×** | | |
| --- | --- | --- |
| EDTA | 15 | g/L |
| ZnSO_4_ · 7 H_2_O | 4.5 | g/L |
| MnCl_2_ · 4 H_2_O | 0.8 | g/L |
| CoCl_2_ · 6 H_2_O | 0.3 | g/L |
| CuSO_4_ · 5 H_2_O | 0.3 | g/L |
| Na_2_MoO_4_ · 2 H_2_O | 0.4 | g/L |
| CaCl_2_ · 2H_2_O | 4.5 | g/L |
| FeSO_4_ · 7H_2_O | 3 | g/L |
| H_3_BO_3_ | 1 | g/L |
| KI | 0.1 | g/L |

| **B. Vitamin solution 1000×** | | |
| --- | --- | --- |
| d-Biotin | 0.05 | g/L |
| Calcium D-(+)-pantothenate | 1 | g/L |
| Nicotinic acid | 1 | g/L |
| Myo-inositol | 25 | g/L |
| Thiamine HCl | 1 | g/L |
| Pyridoxine HCl | 1 | g/L |
| Para-aminobenzoic acid | 0.2 | g/L |


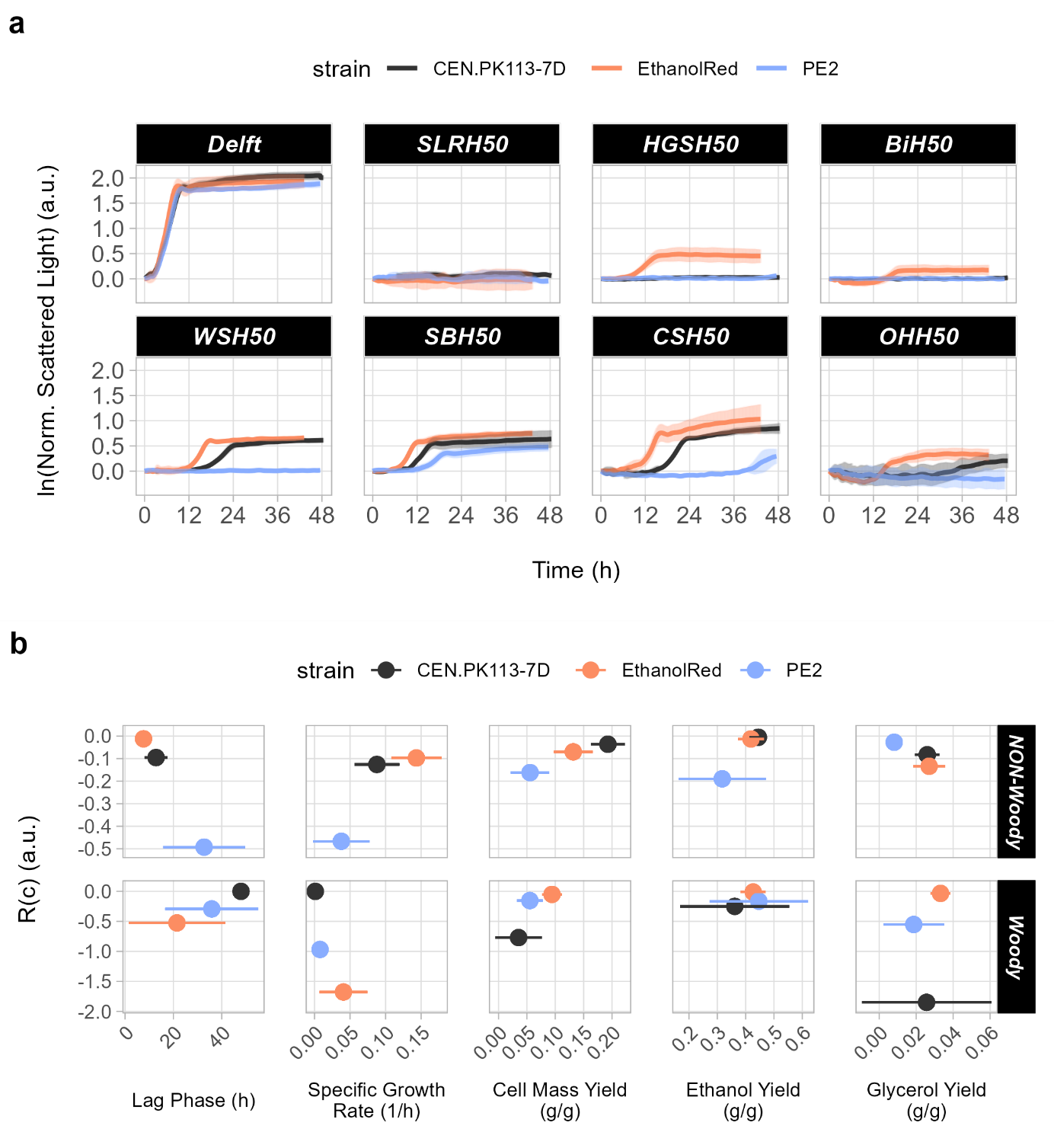
**SUPPLEMENTARY FIGURES**

**Supplementary Figure S1. Growth curves and robustness across conditions in oxygen-limited flask cultivations.** Overview of selected *S. cerevisiae* strains in flask screening under oxygen-limited conditions using different lignocellulosic hydrolysates. (a) Growth curves. (b) Correlation between performance and robustness across conditions, R(c), for five functions (lag phase, specific growth rate, ethanol/glycerol/cell mass yields) based on non-woody (WSH50, SBH50, CSH50, and OHH50) or woody (SLRH50, HGSH50, and BiH50) biomass.


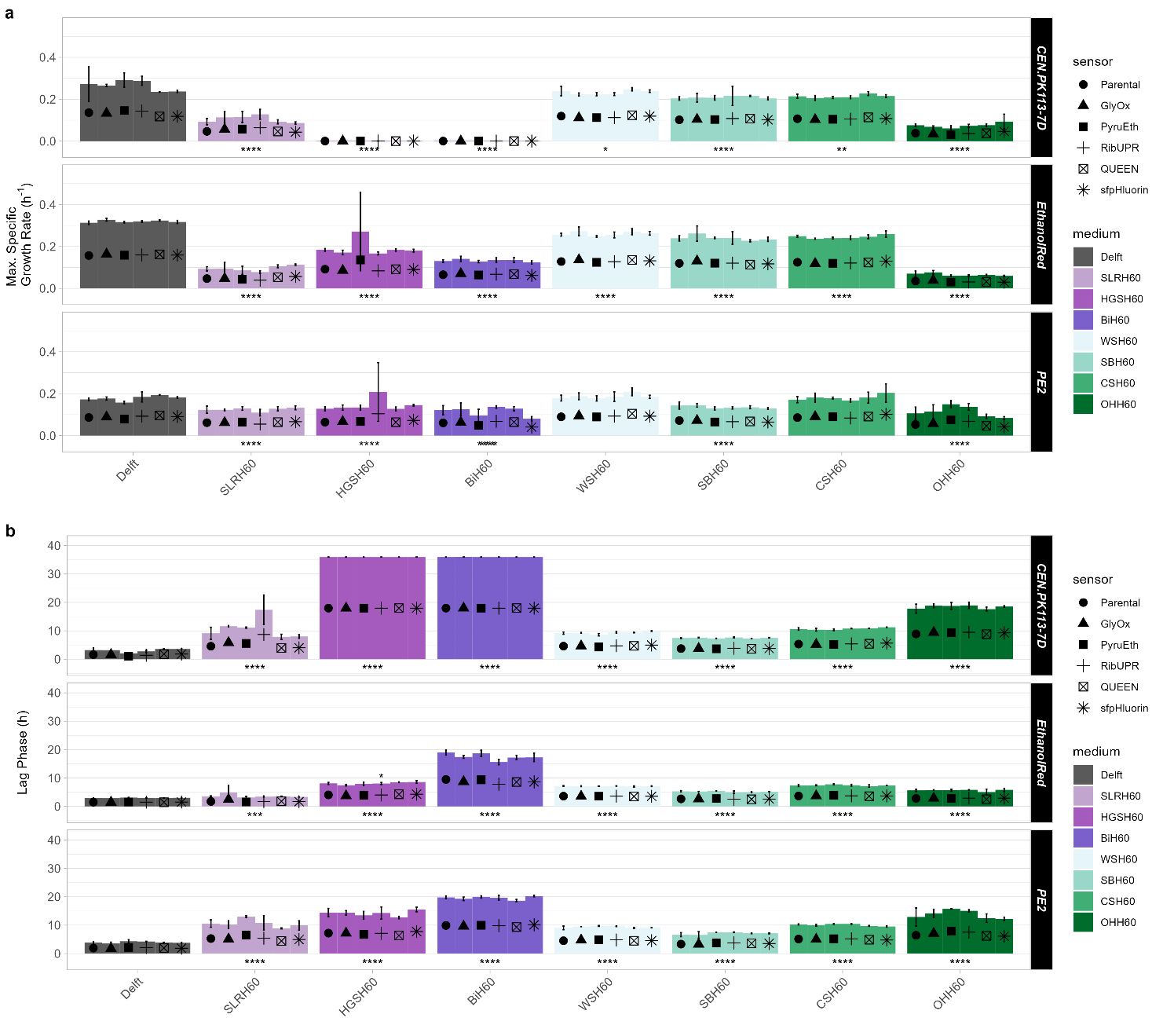


**Supplementary Figure S2. Maximum specific growth rates and lag phases.** Maximum specific growth rates (a) and lag phases (b) of parental and biosensor strains in all tested media. Statistical differences between the biosensor and parental strains are represented above the bar plots; those between the control (Delft) and all other conditions are shown below the bar plots. As no differences in functions were observed between the parental and biosensor strains, all strains (three replicates each) have been used for a more reliable statistical analysis. *p ≤ 0.05; **p ≤ 0.01, ***p ≤ 0.001, and ****p ≤ 0.0001.


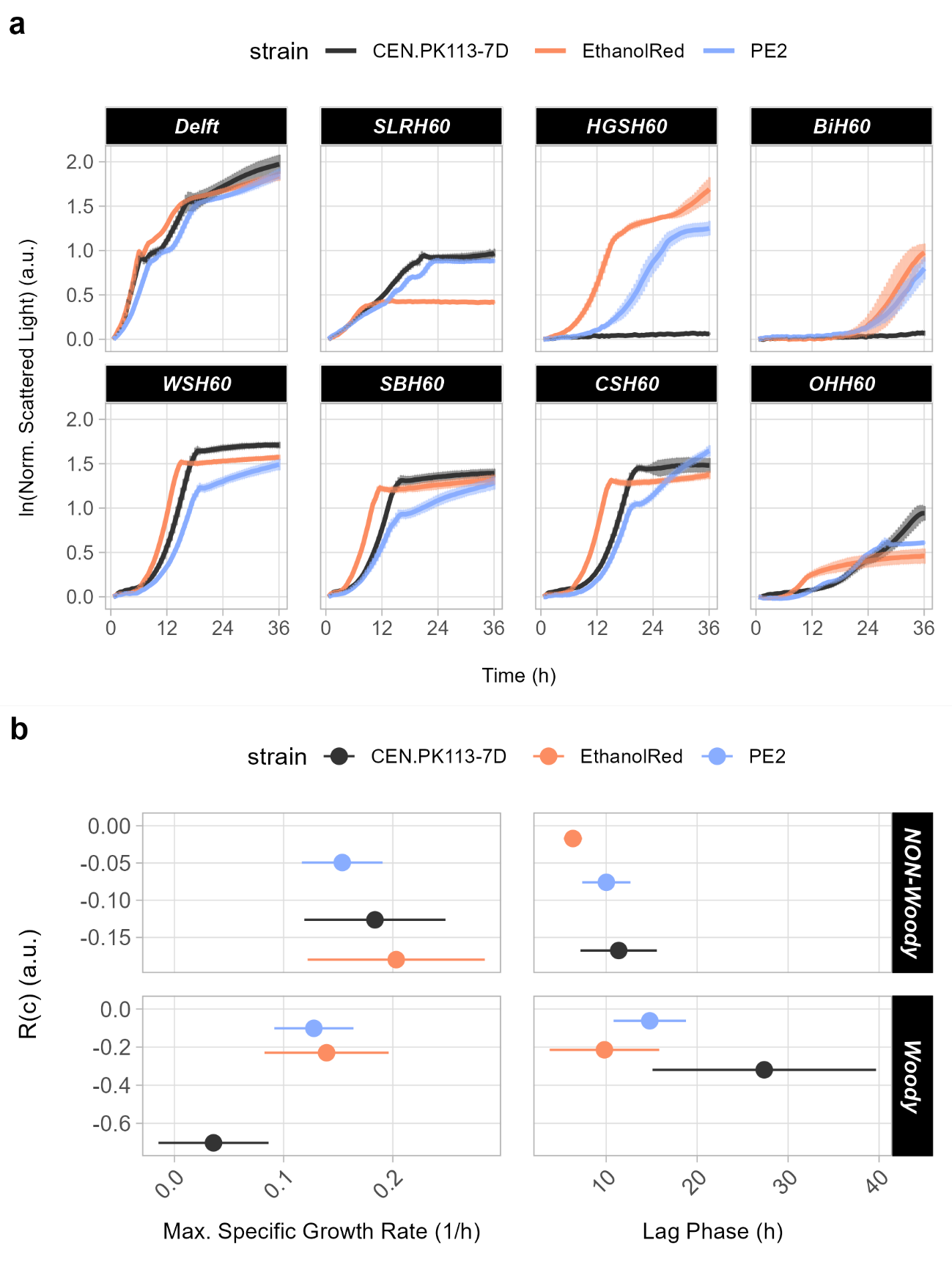
**Supplementary Figure S3. Growth curves and robustness across conditions in BioLector screening.** Overview of selected *S. cerevisiae* strains in flask screening under oxygen-limited conditions using lignocellulosic hydrolysates. (a) Growth curves. (b) Correlation between performance and robustness across conditions for two functions (lag phase and specific growth rate) dividing non-woody (WSH60, SBH60, CSH60, and OHH60) or woody (SLRH60, HGSH60, and BiH60) lignocellulosic hydrolysates.


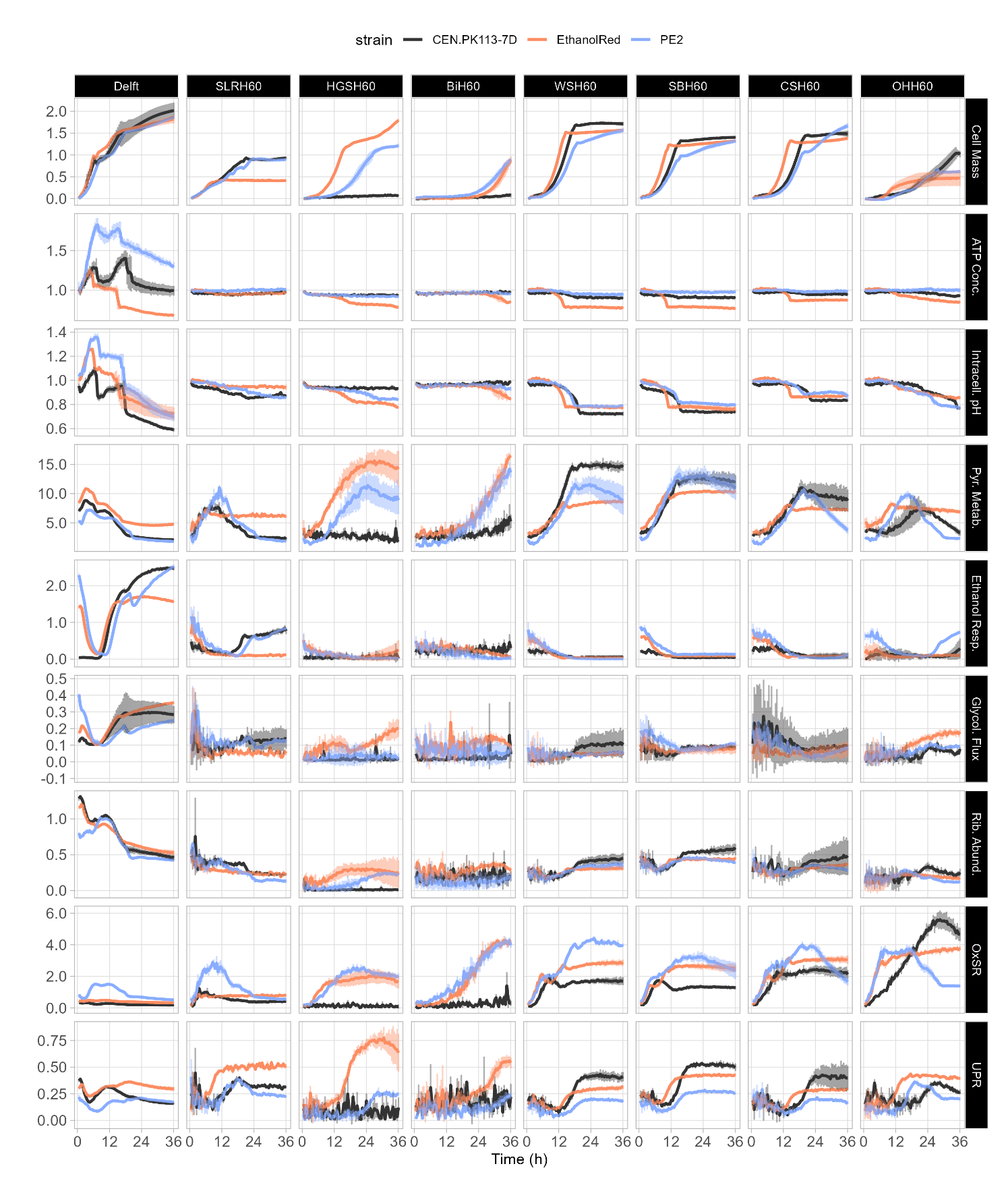


**Supplementary Figure S4. Line plots for intracellular parameters examined via BioLector I screening.** Overview of all line plots for the biosensors used in BioLector screening. Cell mass is represented as the natural logarithm of scattered light, while intracellular parameters are denoted as fluorescence ratios (a.u.).


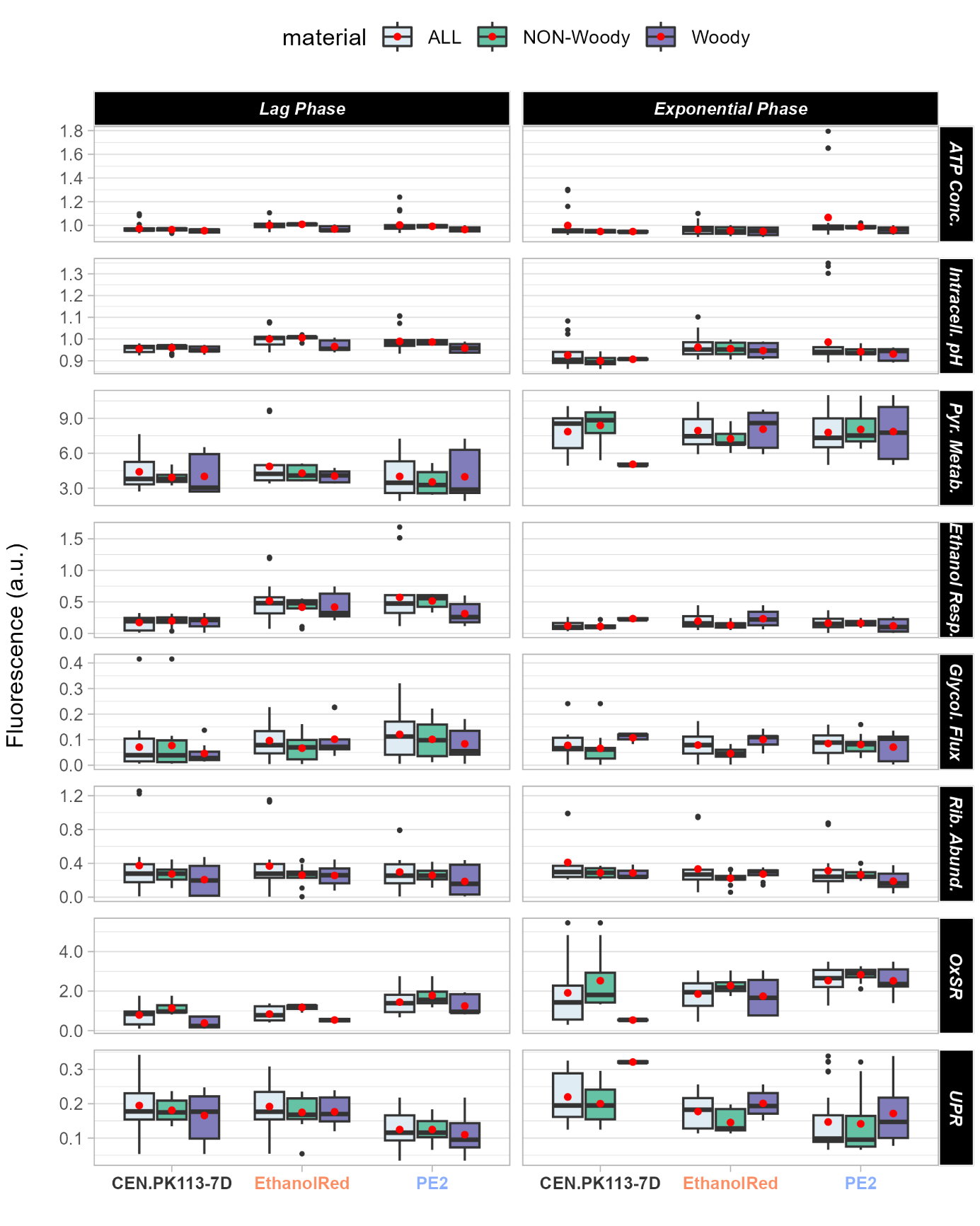
**Supplementary Figure S5. Overview of biosensor outputs categorised by growth phase and medium.** Overview of biosensor output for eight intracellular parameters computed either for the lag phase (left) or exponential phase (right) taking into consideration all tested media (“ALL”), only woody hydrolysates or only non-woody hydrolysates. Red dots identify the mean fluorescent output across all media in that group. Note that for glycolytic flux, the higher the biosensor output the lower the flux.


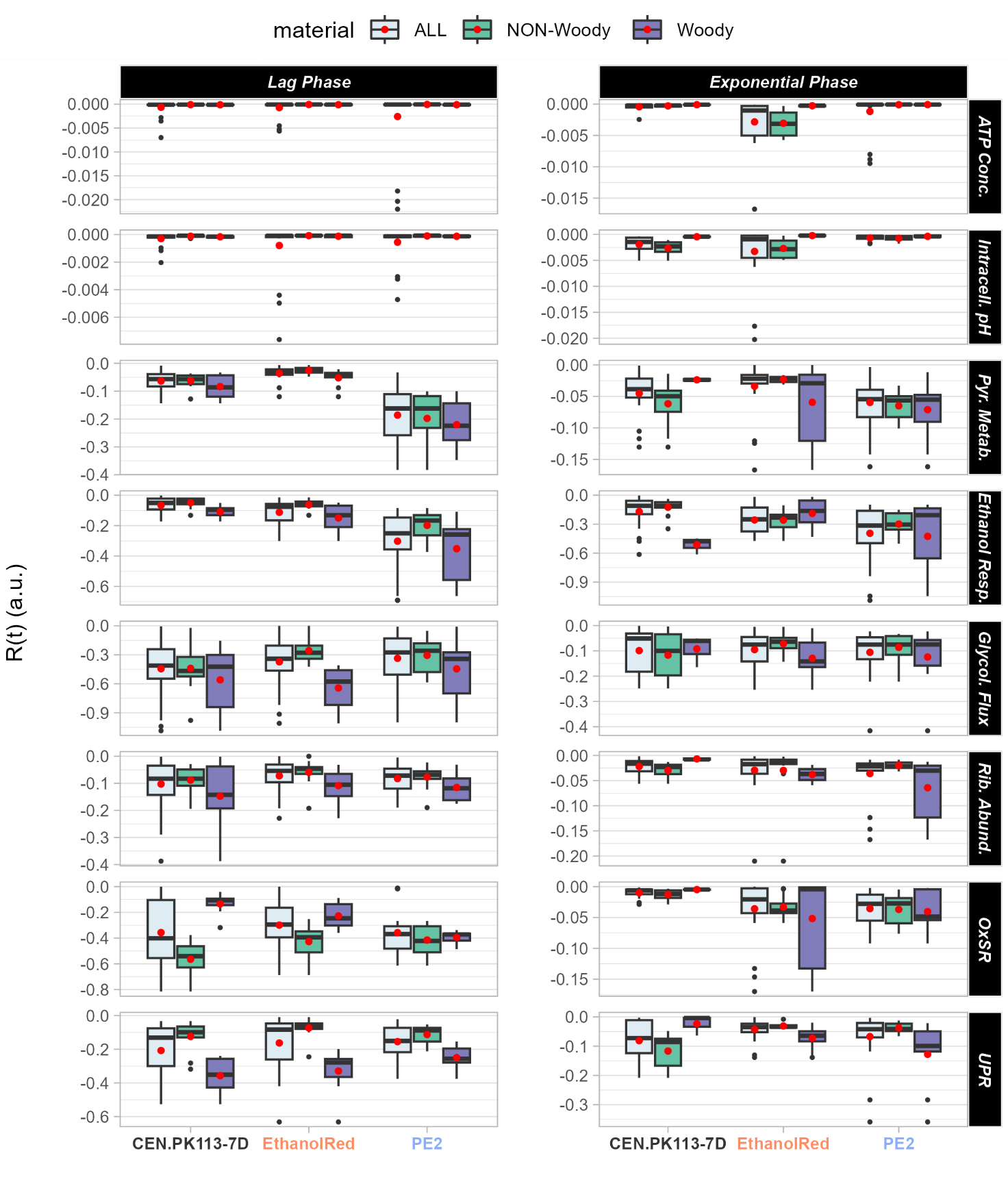
**Supplementary Figure S6. Robustness over time of intracellular parameters categorised by growth phase and medium.** Robustness over time (i.e., the stability of a function over time) for eight intracellular parameters computed either for the lag phase (left) or exponential phase (right). Robustness was computed taking into consideration all tested media (“ALL”), only woody hydrolysates or only non-woody hydrolysates. Red dots identify the mean R(t) across all media in that group.


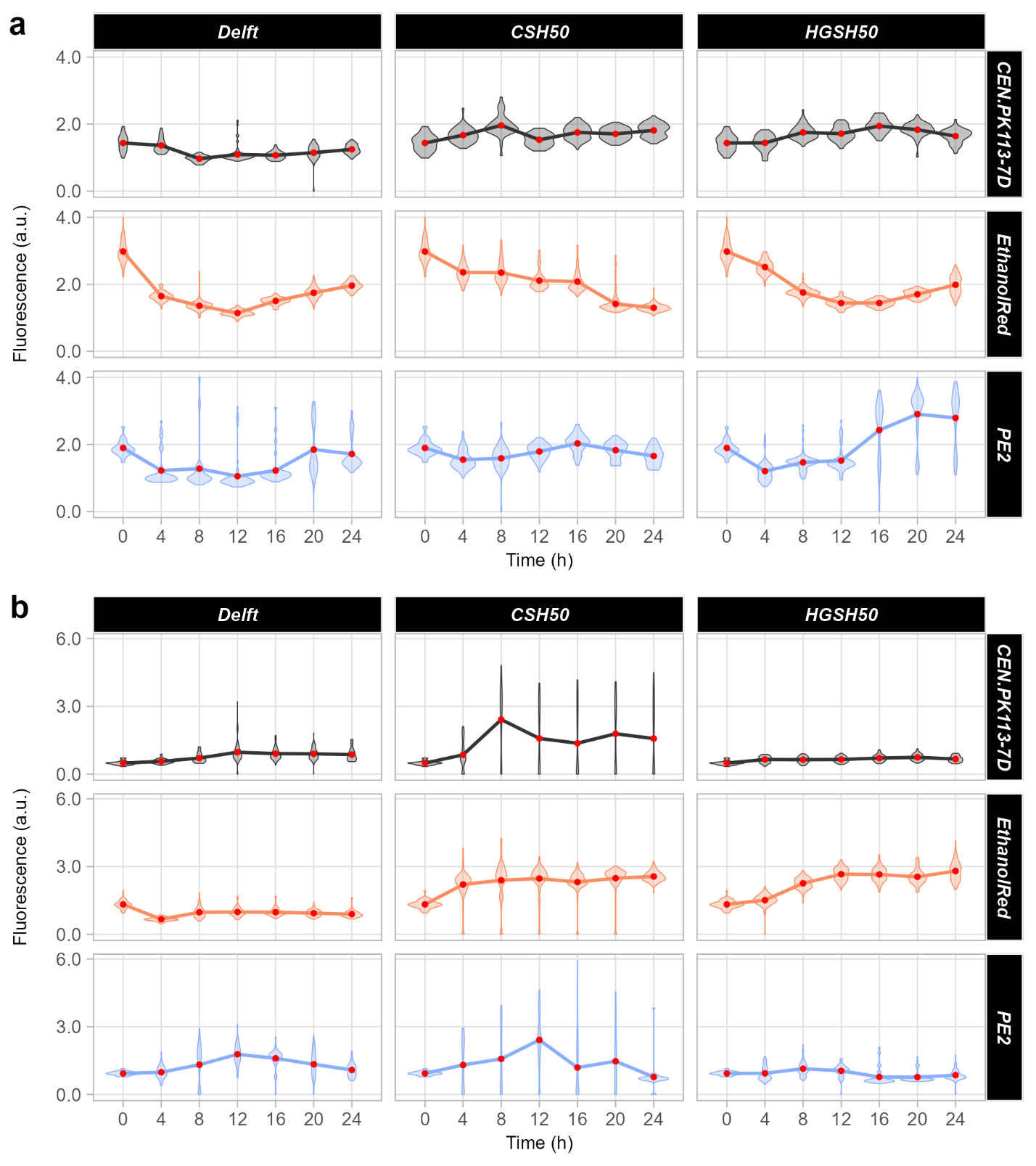


**Supplementary Figure S7. Line plots for GlyOx fluorescence outputs.** Output from the GlyOx biosensor for glycolytic flux (a) and oxidative stress response (b). Violin plots represent the distribution of the intracellular parameter within the cell population at each time point. Red dots represent the mean across all cells. At least 25 cells were analysed for each time point.


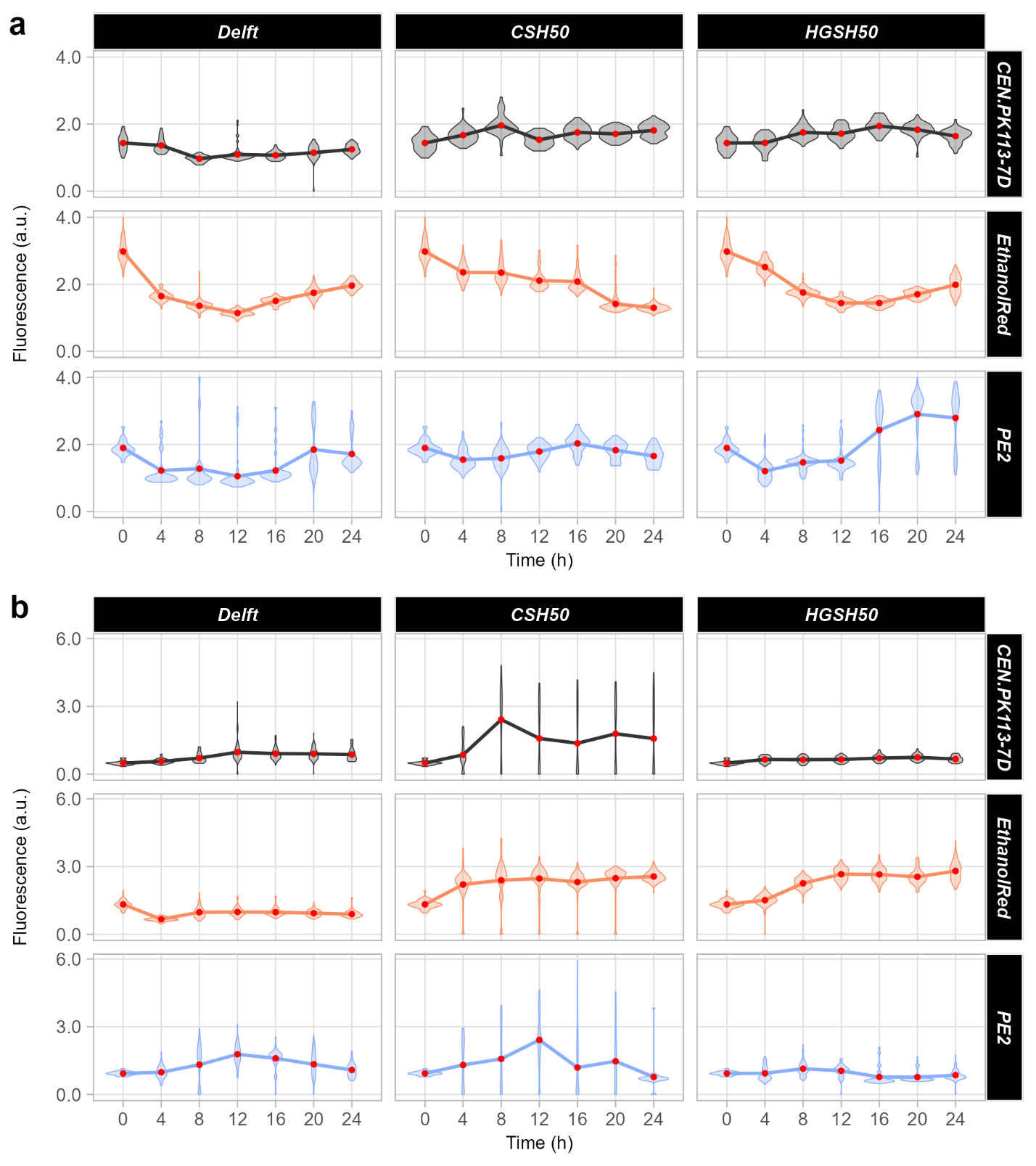


**Supplementary Figure S8. Line plots for RibUPR fluorescence outputs.** Output from the RibUPR biosensor for ribosome abundance (a) and unfolded protein response (b). Violin plots represent the distribution of the intracellular parameter within the cell population at each time point. Red dots represent the mean across all cells. At least 25 cells were analysed for each time point.


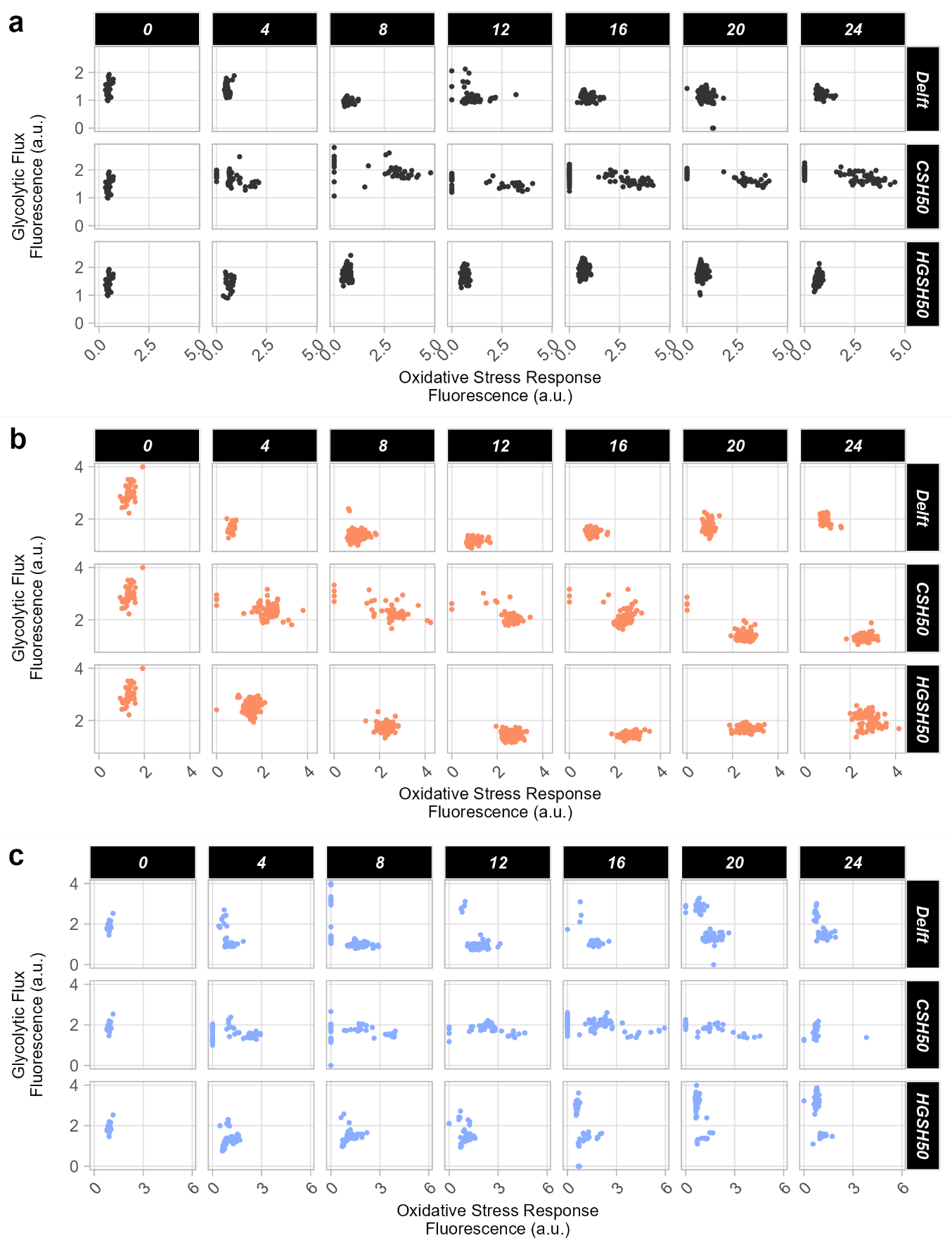
**Supplementary Figure S9. Scatter plots for GlyOx fluorescence outputs.** Scatter plots summarising the correlation between glycolytic flux and oxidative stress response for each cell at each timepoint (0, 4, 8, 12, 16, 20, and 24 h) for CEN.PK113-7D (a), EthanolRed (b), and PE2 (c). Each dot represents a cell. At least 25 cells were analysed for each time point.


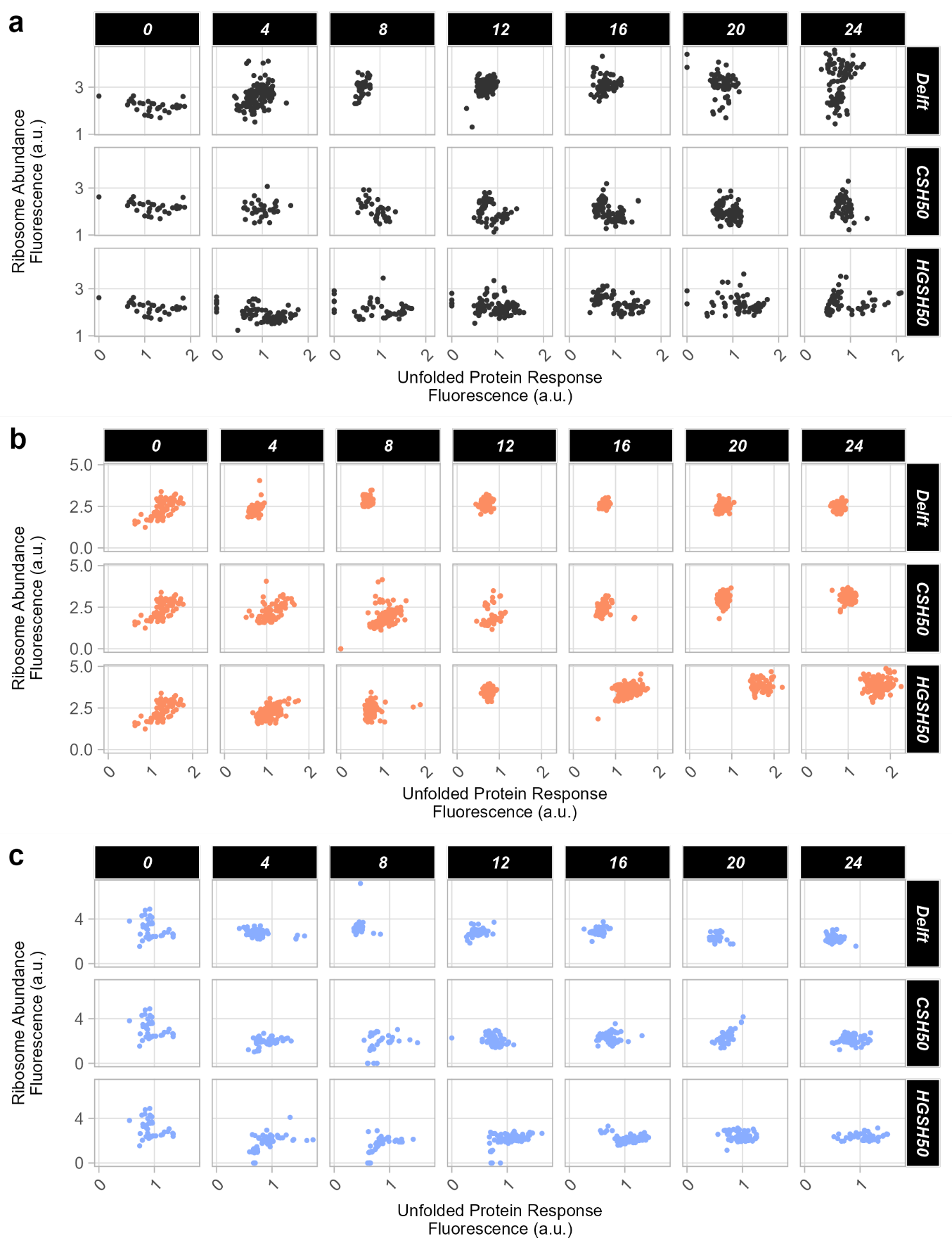
**Supplementary Figure S10. Scatter plots for RibUPR fluorescence outputs.** Scatter plots summarising the correlation between ribosome abundance and unfolded protein response for each cell at each timepoint (0, 4, 8, 12, 16, 20, and 24 h) for CEN.PK113-7D (a), EthanolRed (b), and PE2 (c). Each dot represents a cell. At least 25 cells were analysed for each time point.


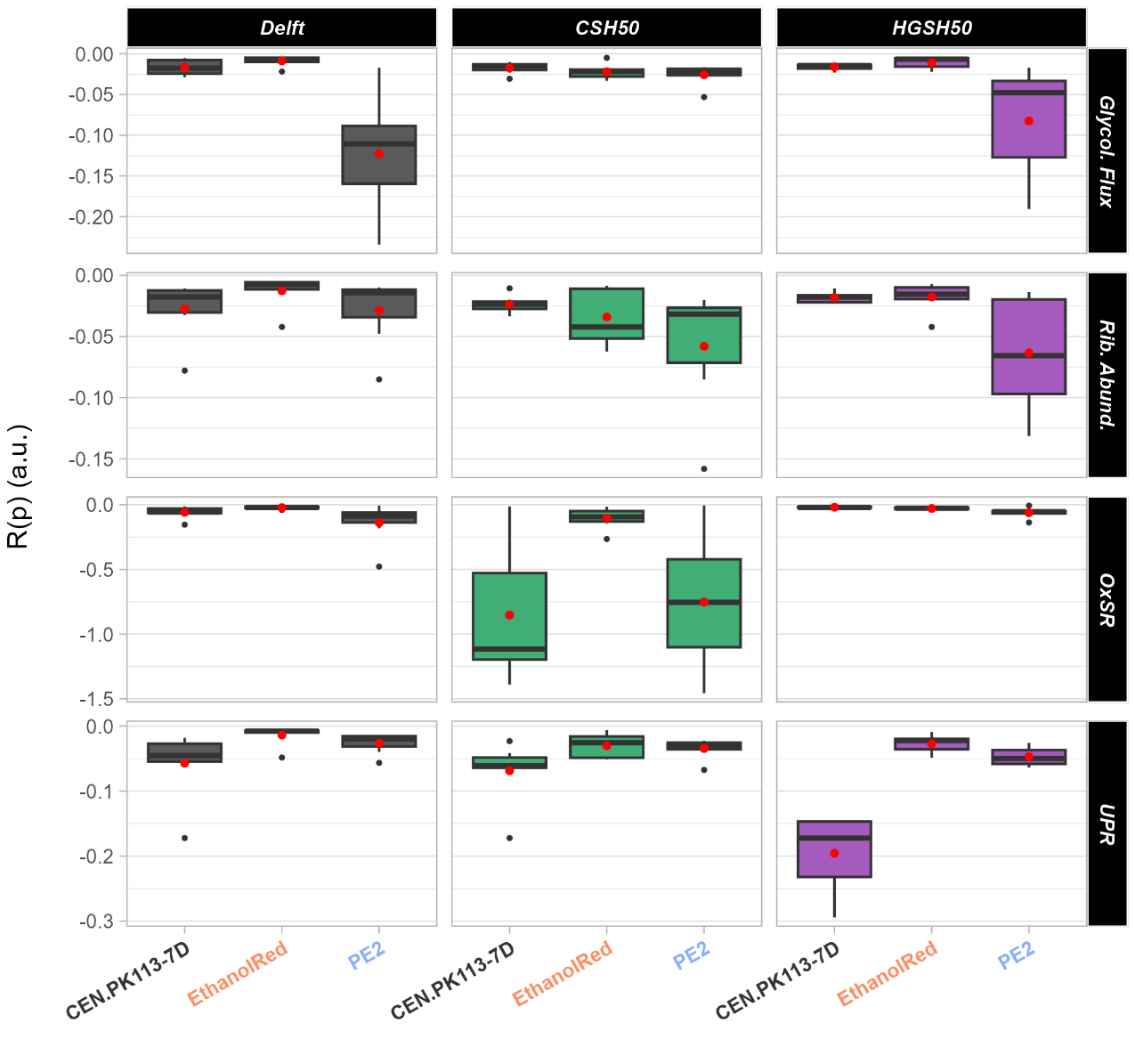


**Supplementary Figure S11. Robustness across populations for intracellular parameters categorised by medium.** Robustness across populations (i.e., how homogeneous a function is within a cell population) for four intracellular parameters (top to bottom): glycolytic flux, ribosome abundance, oxidative stress response, and unfolded protein response. Red dots identify the mean R(p) across all time points for each strain in each medium.

**SUPPLEMENTARY LIST OF ABBREVIATIONS**

WSH: Wheat straw hydrolysate

CSH: Corn stover hydrolysate

OHH: Oat hulls hydrolysate

SBH: Sugarcane bagasse hydrolysate

SLRH: Softwood logging residues hydrolysate

HGSH: High gravity spruce hydrolysate

BiH: Birch hydrolysate

R(c): Robustness across conditions

R(s): Robustness across systems

R(t): Robustness over time

R(p): Robustness across populations

OxSR: Oxidative stress response

UPR: Unfolded protein response

**SUPPLEMENTARY REFERENCES**

1. Knauf M, Moniruzzaman M. Lignocellulosic biomass processing: a perspective. International Sugar Journal. 2004;106(1263):147–50.

2. Rongpipi S, Ye D, Gomez ED, Gomez EW. Progress and opportunities in the characterization of cellulose – an important regulator of cell wall growth and mechanics. Front Plant Sci. 2019 Mar 1;9:410940.

3. Gírio FM, Carvalheiro F, Duarte LC, Bogel-ŁUkasik R. Deconstruction of the hemicellulose fraction from lignocellulosic materials into simple sugars. D-Xylitol: Fermentative Production, Application and Commercialization [Internet]. 2012 [cited 2023 Jul 11];3–37. Available from: https://link.springer.com/chapter/10.1007/978-3-642-31887-0_1

4. Eckardt NA. Role of Xyloglucan in Primary Cell Walls. Plant Cell [Internet]. 2008 [cited 2023 Jul 11];20(6):1421. Available from: /pmc/articles/PMC2483372/

5. Terrett OM, Lyczakowski JJ, Yu L, Iuga D, Franks WT, Brown SP, et al. Molecular architecture of softwood revealed by solid-state NMR. Nature Communications 2019 10:1 [Internet]. 2019 Oct 31 [cited 2023 Jul 11];10(1):1–11. Available from: https://www.nature.com/articles/s41467-019-12979-9

6. Busse-Wicher M, Li A, Silveira RL, Pereira CS, Tryfona T, Gomes TCF, et al. Evolution of Xylan Substitution Patterns in Gymnosperms and Angiosperms: Implications for Xylan Interaction with Cellulose. Plant Physiol [Internet]. 2016 Aug 1 [cited 2023 Jul 11];171(4):2418–31. Available from: https://pubmed.ncbi.nlm.nih.gov/27325663/

7. Stoklosa RJ, Hodge DB. Integration of (Hemi)-Cellulosic Biofuels Technologies with Chemical Pulp Production. Biorefineries: Integrated Biochemical Processes for Liquid Biofuels. 2014 Jan 1;73–100.

8. Marcia MM. Feruloylation in Grasses: Current and Future Perspectives. Mol Plant. 2009 Sep 1;2(5):861–72.

9. Agbor VB, Cicek N, Sparling R, Berlin A, Levin DB. Biomass pretreatment: Fundamentals toward application. Biotechnol Adv. 2011 Nov 1;29(6):675–85.

10. Zoghlami A, Paës G. Lignocellulosic Biomass: Understanding Recalcitrance and Predicting Hydrolysis. Front Chem. 2019 Dec 18;7:478626.

11. Mankar AR, Pandey A, Modak A, Pant KK. Pretreatment of lignocellulosic biomass: A review on recent advances. Bioresour Technol. 2021 Aug 1;334:125235.

12. Jönsson LJ, Martín C. Pretreatment of lignocellulose: Formation of inhibitory by-products and strategies for minimizing their effects. Bioresour Technol. 2016 Jan 1;199:103–12.

13. Liu ZLL. Understanding the tolerance of the industrial yeast Saccharomyces cerevisiae against a major class of toxic aldehyde compounds. Applied Microbiology and Biotechnology. 2018.

14. Liu ZL, Slininger PJ, Dien BS, Berhow MA, Kurtzman CP, Gorsich SW. Adaptive response of yeasts to furfural and 5-hydroxymethylfurfural and new chemical evidence for HMF conversion to 2,5-bis-hydroxymethylfuran. J Ind Microbiol Biotechnol [Internet]. 2004 Sep 1 [cited 2023 Jul 11];31(8):345–52. Available from: https://dx.doi.org/10.1007/s10295-004-0148-3

15. Palmqvist E, Hahn-Hägerdal B. Fermentation of lignocellulosic hydrolysates. II: inhibitors and mechanisms of inhibition. Bioresour Technol. 2000 Aug 1;74(1):25–33.

16. Ullah A, Orij R, Brul S, Smits GJ. Quantitative Analysis of the Modes of Growth Inhibition by Weak Organic Acids in Saccharomyces cerevisiae. Appl Environ Microbiol. 2012;

17. Palma M, Guerreiro JF, Sá-Correia I. Adaptive response and tolerance to acetic acid in Saccharomyces cerevisiae and Zygosaccharomyces bailii: A physiological genomics perspective. Frontiers in Microbiology. 2018.

18. Pampulha ME, Loureiro-Dias MC. Combined effect of acetic acid, pH and ethanol on intracellular pH of fermenting yeast. Applied Microbiology and Biotechnology 1989 31:5 [Internet]. 1989 Oct [cited 2021 Oct 24];31(5):547–50. Available from: https://link.springer.com/article/10.1007/BF00270792

19. Adeboye PT, Bettiga M, Olsson L. The chemical nature of phenolic compounds determines their toxicity and induces distinct physiological responses in Saccharomyces cerevisiae in lignocellulose hydrolysates. AMB Express [Internet]. 2014 Dec 1 [cited 2023 Jul 6];4(1):1–10. Available from: https://amb-express.springeropen.com/articles/10.1186/s13568-014-0046-7

20. Larsson S, Quintana-Sáinz A, Reimann A, Nilvebrant NO, Jönsson LJ. Influence of lignocellulose-derived aromatic compounds on oxygen-limited growth and ethanolic fermentation by Saccharomyces cerevisiae. Applied Biochemistry and Biotechnology - Part A Enzyme Engineering and Biotechnology [Internet]. 2000 [cited 2023 Jul 11];84–86(1):617–32. Available from: https://link.springer.com/article/10.1385/ABAB:84-86:1-9:617

21. Fletcher E, Baetz K. Multi-Faceted Systems Biology Approaches Present a Cellular Landscape of Phenolic Compound Inhibition in Saccharomyces cerevisiae. Front Bioeng Biotechnol. 2020 Oct 14;8:539902.

22. Almeida JRM, Wiman M, Heer D, Brink DP, Sauer U, Hahn-Hägerdal B, et al. Physiological and Molecular Characterization of Yeast Cultures Pre-Adapted for Fermentation of Lignocellulosic Hydrolysate. Fermentation [Internet]. 2023 Jan 1 [cited 2023 Jul 11];9(1):72. Available from: https://www.mdpi.com/2311-5637/9/1/72/htm
